# Nanotubes from Lanthanide-Based Misfit-Layered Compounds: Understanding the Growth, Thermodynamic, and Kinetic Stability Limits

**DOI:** 10.1021/acs.chemmater.4c00481

**Published:** 2024-04-30

**Authors:** M. B. Sreedhara, Azat Khadiev, Kai Zheng, Simon Hettler, Marco Serra, Ivano E. Castelli, Raul Arenal, Dmitri Novikov, Reshef Tenne

**Affiliations:** † Solid State and Structural Chemistry Unit, Indian Institute of Science, Bengaluru 560012, India; ‡ Deutsches Elektronen-Synchrotron DESY, Notkestr. 85, 22607 Hamburg, Germany; § Department of Energy Conversion and Storage, Technical University of Denmark, DK-2800 Kgs. Lyngby, Denmark; ∥ Instituto de Nanociencia y Materiales de Aragon (INMA), CSIC-Universidad de Zaragoza, 50018 Zaragoza, Spain; ⊥ Laboratorio de Microscopias Avanzadas (LMA), Universidad de Zaragoza, 50018 Zaragoza, Spain; # Dipartimento di Scienze Chimiche e Geologiche, Università di Modena e Reggio Emilia, Via G. Campi 103, 41125 Modena, Italy; ∇ ARAID Foundation, 50018 Zaragoza, Spain; ○ Department of Molecular Chemistry and Materials Science, Weizmann Institute of Science, Rehovot 7610001, Israel

## Abstract

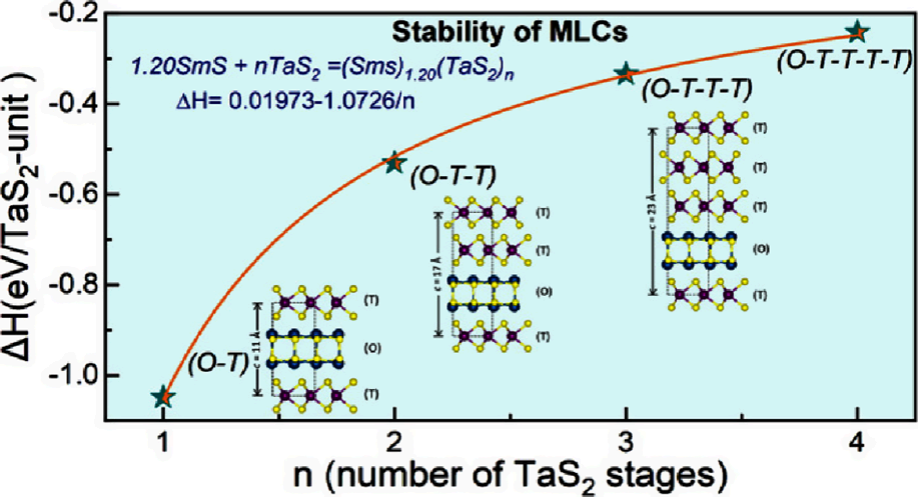

Gaining insights into the kinetics and the thermodynamic limits of nanostructures in high-temperature reactions is crucial for controlling their unique morphology, phase, and structure. Nanotubes from lanthanide-based misfit-layered compounds (MLCs) have been known for more than a decade and were successfully produced mostly via a chemical vapor transport protocol. The MLC nanotubes show diverse structural arrangements and lattice disorders, which could have a salient impact on their properties. Though their structure and charge transfer properties are reasonably well understood, a lack of information on their thermodynamic and kinetic stability limits their scalable synthesis and their applicability in modern technologies. In this study, the growth, thermodynamic stability, and decomposition kinetics of lanthanide-based misfit nanotubes of two model compounds, i.e., (LaS)_1.14_TaS_2_ and (SmS)_1.19_TaS_2_ are elucidated in detail. The nanotubes were carefully analyzed via atomic resolution electron microscopy imaging and synchrotron-based X-ray and electron diffraction techniques, and the information on their morphology, phase, and structures was deduced. The key insights gained would help to establish the parameters to explore their physio-chemical properties further. Furthermore, this study sheds light on the complex issue of the high-temperature stability of nanotubes and nanostructures in general.

## Introduction

Owing to their large surface-to-volume ratio, nanostructures are, in general, metastable and highly reactive compared to their bulk analogs. Generally speaking, the thermodynamic stability of the nanostructures and the kinetics of their decomposition were studied very sparsely. Efforts in this direction could bring insights into solid-state diffusion, entropy vs. enthalpy terms, chemical affinity, and the high-temperature metastability of nanophases compared to the bulk. In addition, a careful analysis of the kinetics and the thermodynamics via synthetic parameters is pivotal to scaling up the synthetic process to maintain the unique morphology and homogeneity of the nanocrystalline phase.

Inorganic compounds with layered structures, including transition metal dichalcogenides (TMDCs), like WS_2_, are known to form fullerene-like nanostructures (IF) and nanotubes (INT).^1−4^ IF nanoparticles (NPs) of WS_2_ and nanotubes thereof were produced in large quantities more than a decade ago and their growth mechanism is quite well understood.^5^ Various other kinds of nanotubes and IF NPs from different compounds with layered structures have been studied experimentally and in silico.^6^ In an attempt to elucidate the stability of these nanophases, the thermal degradation of fullerene-like (IF) NPs of WS_2_, MoS_2_, and NbS_2_ were investigated and compared to the corresponding micron-sized platelets in both inert and ambient atmospheres.^7^ As anticipated, the IF NPs were found to be somewhat less stable against high-temperature decomposition in both inert and oxidizing atmospheres compared with the bulk platelets.

**Figure 1:**
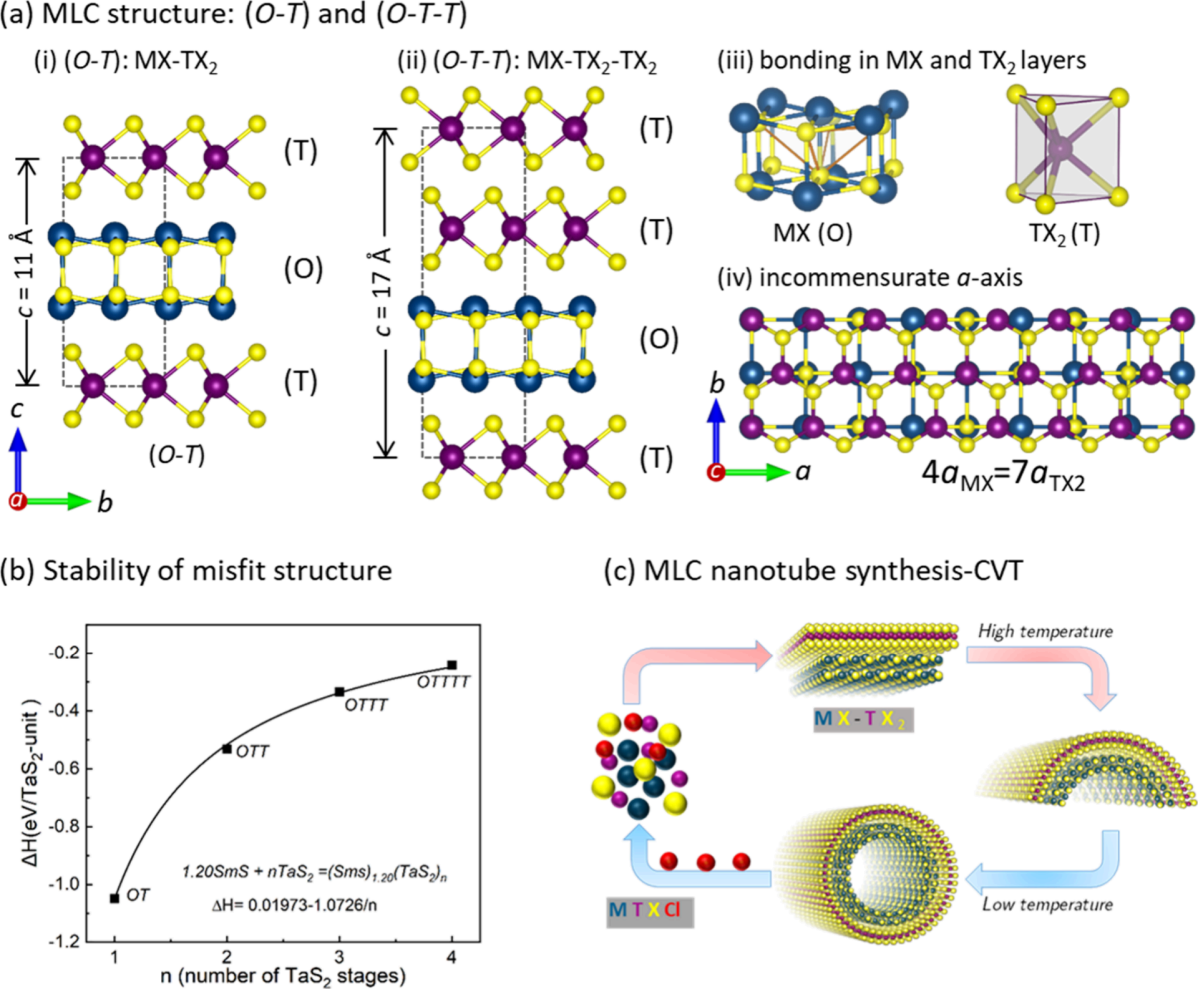
(a) Illustration of stage-1 (*O*–*T*) and stage-2 (*O*–*T*–*T*) MLC structures projected in the *bc* plane: The distorted rocksalt MX and hexagonal TX_2_ are stacked alternately as periodic superstructures along the *c*-direction. The pseudo-octahedral (*O*) and trigonal prismatic (*T*) coordination of the rocksalt and hexagonal units are shown. The incommensurate lattice along crystallographic *a* direction is shown. (b) Stability of the misfit structure as a function of number of *T* layers in a single misfit unit cell calculated using DFT simulations. (c) Schematic depiction of the growth mechanism of MLC nanotubes via CVT reaction, the transport agent chlorine is represented in red solid spheres.

Among the classes of nanotubes, those from misfit-layered compounds (MLC) are of particular interest. MLC are incommensurate and nonstoichiometric structures with the general formula (MX)_*1+y*_(TX_2_)_*n*_, where M = rare-earth (*Ln*), Pb, Bi, Sn, etc., T = Ta, Nb, Ti, V, Cr, and X = S, Se. MX is a (distorted) rocksalt layer also designated as *O*, and the hexagonal TX_2_ layer is designated as *T*. The value of *n* indicates the stage, i.e., *n* = 1 is stage-1 (*O–T*), n = 2 is stage-2 (*O*–*T*–*T*) MLC (Figure 1a), and so on. The incommensuration parameter *1+y* is the mismatch parameter (stoichiometric deviation) along the crystallographic *a* direction. The unit cell of an approximant within an MLC incorporates  units of the TaS_2_ lattice along the *a*-axis with the unit cell of the MX lattice. The *c*-axis, representing the interlayer spacing, is shared between the two crystalline layers. The structural asymmetry and strong coupling between the *O* and *T* layers in MLC suggest that they are prone to fold and form nanotubes. Indeed, MLC nanotubes were discovered by serendipity^8−10^ and have been investigated for more than a decade now.^11^ In fact, the driving force for the formation of MLC nanotubes (local stability) is attributed to the concerted action of the incommensurate structure, which forces the layers to bend, and the seaming of the dangling bonds at the periphery of the nanosheets.^4^ MLC nanotubes (and nanoscrolls thereof) are believed to be generically metastable at elevated temperatures and hence would be expected to convert into the bulk MLC (flakes) or their individual binary constituents upon heating and long annealing periods. Bulk MLC compounds have been synthesized by various methods such as high-temperature solid-state reactions, mechanochemical reactions,^12^ flux growth, and chemical vapor transport (CVT).^13,14^ Among the known chalcogenide MLCs, rare-earth-based (*Ln*-) misfits constitute a distinct family of compounds. The nanotubes of *Ln*-misfit compounds were successfully prepared via a CVT protocol (Figure 1c). Though the established CVT procedure yields nanotubes in reasonable quantities and purity, efforts to unravel the growth mechanism and optimize the nanotube yield have been limited so far. The abundance and the aspect ratio of nanotubes vary vastly with the *Ln* atom present in the rocksalt unit.^13,15^ From previous studies, it is known that alloying the *Ln* lattice site, i.e., formation of Ln_1*x*_Ln_2(1-x)_S-TaS_2_ and randomized mixing of the two *Ln* atoms (increased entropy) of the rocksalt unit, increases the nanotube yield.^14,16^ Since most of the misfit nanotubes grown so far were obtained at a single definite temperature and time (850 °C, 6 h), it is hard to derive any information regarding their growth kinetics and stability.

Here, using the two *Ln*-based misfit model compounds, i.e., (LaS)_1.14_TaS_2_ and (SmS)_1.19_TaS_2_, the role of the transport agent, temperature, reaction time, and quenching rate on the nanotubes’ yield was analyzed, first. This analysis produced the optimized parameters for the growth of the MLC nanotubes in relatively high purity and respectable amounts (a few tens of milligrams per batch). Further, MLC nanotubes prepared according to the optimized parameters at high yields were subjected to a high-temperature annealing process, thereby gaining insight into their prospective stability. The growth of the nanotubes and the morphological changes with respect to temperature and (annealing) time were followed using scanning and transmission electron microscopy, first. The detailed structural evolution and transformation of (*O*–*T*) to a mixture of (*O*–*T···O*–*T*–*T*) was studied further using laboratory and synchrotron-based X-ray diffraction (XRD) techniques. Owing to their higher stability at ambient conditions compared to (LaS)_1.14_TaS_2_, (SmS)_1.19_TaS_2_ nanotubes were characterized extensively. It was found that the abundance and aspect ratio of the (SmS)_1.19_TaS_2_ tubes grow upon increasing the reaction temperature from 800 to 825 °C and the reaction time from 2 to 4 h. Subsequently, they gradually disappear at higher temperatures or longer reaction times. The products of the long-term annealing were analyzed to elucidate the degradation mechanism of the nanotubes. Deciphering the details for the optimized growth of *Ln*-based MLC nanotubes and their stability parameters at high temperatures is anticipated to help researchers reproduce this family of nanotubes in higher abundance and investigate their many-body physical properties.

## Results and Discussion

Lanthanide-based MLCs (LnS*–*TaS_2_) are incommensurate layered superstructures with alternating layers of LnS (rocksalt or *O*) unit and hexagonal TaS_2_ (*T*) units (Figure 1a). The incommensuration along the crystallographic *a* direction induces a misfit between the MX and TX_2_ unit, and the parameter *y* = 2a_*T*_/a_*O*_-1 varies between 0.08 ≤ *y* ≤ 0.28. The structural stability in these compounds is attributed to strong charge transfer from the LnS sublattice to partially filled *5dz*^*2*^ states of Ta. The stability of MLC compounds with different stacking orders (stages 1–4) was confirmed by quantum mechanical calculations (Figure 1b). Stage 1 compound exhibits the highest charge transfer, which subsequently decreases with increasing value *n,* the number of TX_2_ layers. As a consequence, depletion of the LnS unit destabilizes the MLC, which leads to phase separation into the binary compound. The calculated stability, by means of density functional theory (DFT), as a function of the increasing number of hexagonal units is shown in Figure 1b. The order of stability is *O–T* > *O–T–T* > *O–T–T–T*>··· and so on. Obviously, this calculation is valid at low temperatures and does not pertain to high temperatures where a large entropic contribution to the free energy of the reaction becomes significant. MLC nanotubes containing a large fraction of *O–T–T–T* repeat units were not found, so far.^17^ This omission was attributed to the absence of the *O–T* interface for the middle *T* layer, which prevents effective charge transfer and lowers the overall stability. The various reaction parameters conducive to the growth of (SmS)_1.19_TaS_2_ nanotubes were studied first in this work, resulting in the synthesis of copious amounts of such nanotubes. Subsequently, the kinetic and thermodynamic stability of such nanotubes were studied at elevated temperatures.

### Synthesis of Ln-Based MLC Nanotubes

(LaS)_1.14_TaS_2_ and (SmS)_1.19_TaS_2_ nanotubes were prepared via a CVT protocol (Figure 1c, refer to the Experimental Section). CVT growth is a catalytic process and is induced by a volatile gas, like chlorine. Presumably, the growth of the multiwall chiral nanotubes occurs atom-by-atom analogous to the growth of chiral structures like tellurium nanowires via screw dislocations.^18^ The reaction parameters such as the transport agent, reaction temperatures, and times were varied to get high yields of nanotubes. As obtained, MLC nanotubes were subjected to a high-temperature annealing process above 1000 °C to understand the kinetic and thermodynamic stabilities of these nanotubes.

### Role of the Transport Agent

Chalcogenide-based MLC single crystals and flakes were grown using various transport agents such as I, Br, and Cl. However, there is no clear evidence in the literature of the specific reasons for adopting different transport agents. Though bromine and iodine are commonly used as transport agents for the growth of a large variety of TMDCs, in the case of MLCs, Cl is widely used for the growth. In a search for the best transport agent, several reactions of (SmS)_1.19_TaS_2_ MLC at optimized growth conditions for a high yield of nanotubes (825 °C, 4 h) were carried out using different transport agents (see Table S1). All three transport agents (I, Br, and Cl) yield the misfit structures, but MLC nanotubes were obtained with chlorine and bromine, whereas iodine did not yield any nanotubes/scrolls structures (Figures 1a and S1). Though bromine appears to be a very good transport agent for producing misfit nanotubes, considerable amounts of bromine (>11 at %) were found to be occluded in the nanotubes’ lattice (Figure S1e), hence it would be expected to largely affect their properties. In the case of chlorine, energy-dispersive X-ray spectroscopy (EDS) SEM scans of large specimens showed a negligible quantity of chlorine to be present in the misfit structures. Thus, chlorine seems to be a good choice for preparing misfit structures and their nanotubes. Another important observation made in these reactions is that unlike the regular CVT process, the MLC flakes and nanotubes were found to form at the hotter zone, and no mass transport to the colder regime was observed. A possible reason for this observation could likely be that the misfit structures are energetically more stable at these temperatures compared to their binaries and/or the temperatures might not be sufficient for mass transport. This observation does not mean that CVT reaction is excluded, but rather that it occurs locally under an appreciably smaller temperature gradient of a few degrees. Reactions carried out without the transport agent did not yield the misfit structure at all, and such reactions ended up in a mixture of binary phases comprising 3*R*-TaS_2_ with flakes morphology (Figure S2a) and orthorhombic Sm_2_S_3_ which is further confirmed by XRD (Figure S2c). These results entail that a CVT mechanism through a gas phase reaction is a prerequisite for the formation of the nanotubes and temperature differences as small as a few degrees are sufficient for the effective catalytic growth of the nanotubes.

### Role of Reaction Temperature and Time

**Figure 2:**
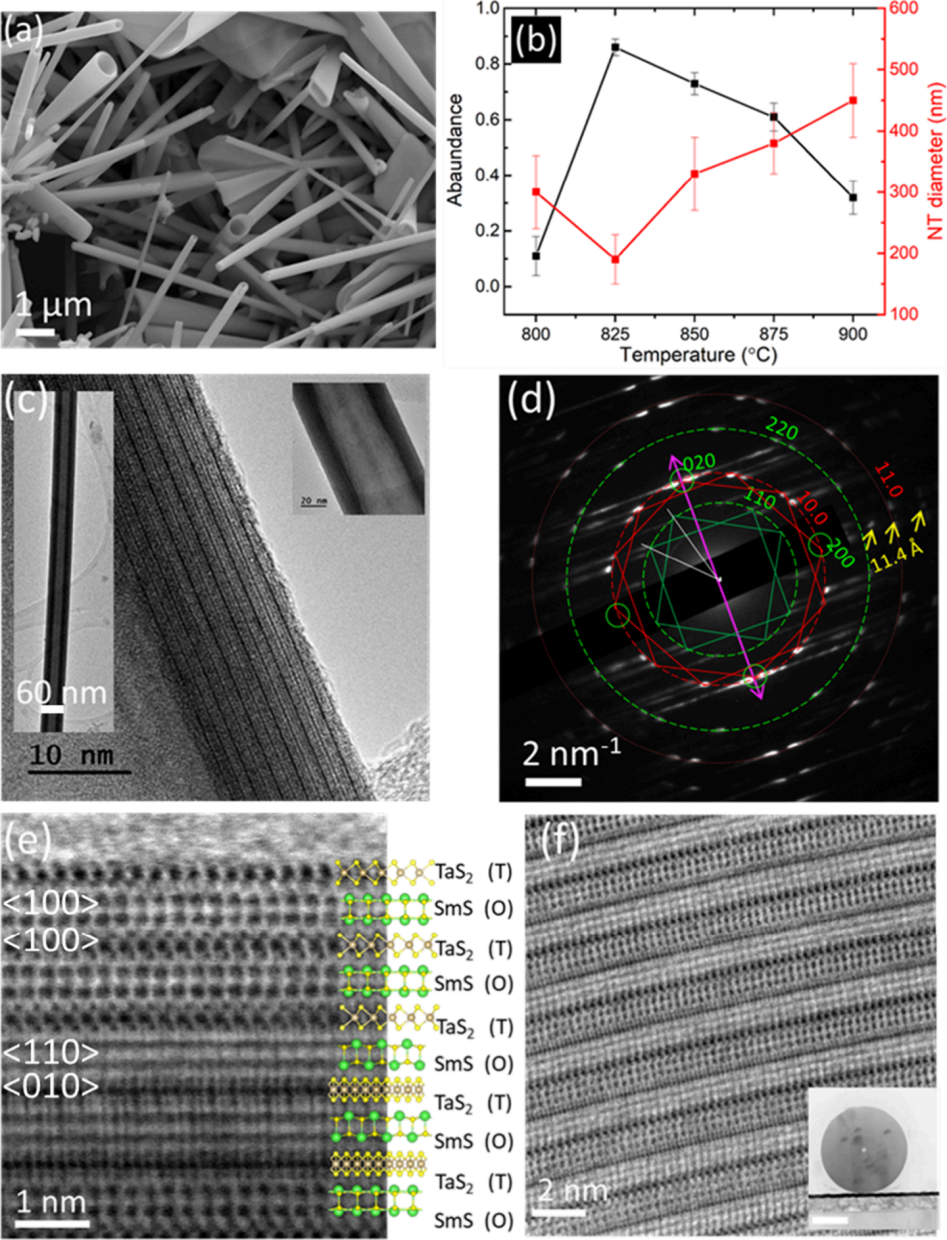
Structural and morphological analysis: (a) A typical SEM micrograph of (SmS)_1.19_TaS_2_ nanotubes obtained at 825 °C using Cl as a transport agent. (b) Relative abundance and variation of the mean diameter of (SmS)_1.19_TaS_2_ nanotubes as a function of growth temperatures obtained by a statistical analysis of several SEM images from each product. The error bars show the deviation of the value within the different (8–10) images analyzed per each kind of sample. (c) Low (inset) and high magnification TEM image of (SmS)_1.19_TaS_2_ nanotube comprising 12 misfit layers which are arranged in a regular (*O–T*) and (*O–T*)′ superstructure. (d) Electron diffraction pattern obtained from the nanotube shown in panel (c); the nanotube growth direction is marked by a pink double arrow. The diffraction patterns corresponding to the rocksalt SmS and trigonal prismatic TaS_2_ are marked with green squares and red hexagons, respectively. The two folding vectors separated by 30° between the misfit layers are marked with white lines and are in accordance with the HRTEM image. The (200) and (020) Bragg planes of rocksalt SmS are marked in solid green circles, and the common *c*-axis reflections are shown in yellow arrows. (e) Atomic resolution STEM (BF) image of a nanotube, each rocksalt SmS, and trigonal prismatic TaS_2_ layers are marked, and the atomic models are overlaid on the image for clarity. (f) STEM image of a focused ion beam (FIB) cut lamella of the nanotube (inset) and the corresponding atomic resolution STEM (BF) image of a portion of the lamella showing the regular arrangement of the misfit lattice with the (*O–T*) superstructure. Each misfit layer is rotated by 30° with respect to the adjacent layers, i.e., (*O–T*)(*O–T*)′ superstructure, also seen in panels (c,e).

To evaluate the right temperature and time profiles for nanotube growth, a series of reactions were carried out between 800 and 975 °C and a reaction period of 1–16 h (Tables S1 and S2). A typical SEM image of (SmS)_1.19_TaS_2_ nanotubes grown at 825 °C is shown in Figure 2a. The detailed analysis of the nanotube abundance and the evolution of their size are presented in Figures 2b and S3. The highest abundance of nanotubes for (SmS)_1.19_TaS_2_ and (LaS)_1.14_TaS_2_ were observed at 825 and 875 °C for a reaction time of 4 h, respectively. The disparity in the temperature for the optimized yield of nanotubes of the two systems can be attributed to the entropy of the reaction and the misfit strain between the rocksalt and hexagonal lattice. The strain between SmS and TaS_2_ is estimated to be 3.8% (in the *a*-axis direction), whereas for LaS it is 2.3%. The higher mismatch (strain) in the samarium-based MLC would lead (SmS)_1.19_TaS_2_ to roll into nanotubes under milder conditions than (LaS)_1.14_TaS_2_. With increasing temperature, the flakes would grow faster as infinite layers thereby reducing the chance for nanotube formation, which is true for both systems. In addition to the nanotubes and flakes, scrolls formed by rolling the sheets were found in appreciable amounts. It is quite difficult to separate the nanotubes and scrolls from the flakes by any known technique, considering the sensitivity of the samples to ambient conditions. It is quite understandable that the formation of scrolls would result from the folding of platelets with large lateral dimensions. Contrarily, the catalytic growth of the nanotubes proceeds, most likely, atom-by-atom much like the growth of chiral nanostructures like tellurium nanowires via screw dislocation.^18^ Further experimental and theoretical work is required to prove the mechanism of the formation of nanotubes and scrolls. Interestingly, the average diameter of the nanotubes (Figure 2b) and the size of the flakes increases with temperature, which correlates with their reduced abundance. The tubes grown above 900 °C show an average diameter exceeding 0.5 μm with lengths of several tens of micrometers, and their abundance goes down drastically. Hence, it is important to maintain the temperature window between 800 and 900 °C for the growth of lanthanide-based MLC nanotubes with appreciable yield and within a narrow range of diameters. The occurrence of an optimum temperature for the growth of MLC nanotubes is not surprising. As the temperature is increased, the nucleation density of the nanotubes’ seeds increases, and the CVT reaction itself is hastened. However, at higher temperatures, Ostwald ripening is set on, with the larger diameter tubes growing at the expense of the smaller ones, which gradually disappear. Eventually, at high enough temperatures, the entropy term favors the production of binary compounds at the expense of the ternary MLC tubes/flakes.

The reaction time also plays a significant role in obtaining nanotubes with a high abundance. Upon increasing the reaction time, the nanotubes are gradually converted into microtubules and flakes (Figure S4). The reaction time duration between 2 and 4 h appears to be the optimum time to get the nanotubes in appreciable yield and high aspect ratio. A reaction time of less than 2 h is not sufficient to convert the entire precursor into a misfit structure ending up with TaS_2_ impurities. In addition to time and temperatures, the quenching rate of the reaction influences the abundance of the nanotubes and their aspect ratio. In the regular process, the reaction mixture was retracted from the hot zone and naturally cooled to room temperature. Quenching in water and liquid nitrogen (LN_2_) was also attempted. Quenching in liquid nitrogen yields very fine and thin misfit nanotubes (Figure S5), but additionally, the powder also contains a significant amount of TaS_2_ and unreacted Ta as byproduct. So, for further studies, the regular quenching method has been followed.

To evaluate the local structure, (SmS)_1.19_TaS_2_ nanotubes produced at 825 °C were studied in detail by high-resolution (scanning) TEM (HR(S)TEM) imaging and electron diffraction (ED). A low-magnification TEM image in the inset of Figure 2c shows a nanotube with a constant diameter of 60 nm. The HRTEM images reveal the alternate stacking of TaS_2_ and SmS layers corresponding to a misfit structure. The darker lines correspond to three-atom thick trigonal prismatic TaS_2_ layers, and the adjacent brighter layers are two-atom thick rocksalt SmS layers, respectively. The nanotube constitutes 12 layers of TaS_2_ and 12 layers of SmS which are alternately stacked along the *c*-direction. The nanotube growth direction coincides with the crystallographically commensurate *b*-direction, and the misfit *a*-direction is along the circumference of the nanotube (refer to the orthohexagonal cell in Figure 1a). It is evident from the HRTEM image and ED that each misfit (*O*–*T*) layer is rotated by 30° with respect to the adjacent ones forming a supercell with a large *c*-plane parameter of 22 Å, which is quite common in these compounds.^19^ The electron diffraction pattern in Figure 2d shows a streaky pattern, which is a hallmark of the nanotube geometry. The reflections from the rocksalt SmS and hexagonal TaS_2_ are marked in green and red color, respectively. A set of eight reflections (two folding vectors) from (110) planes of SmS and 12 reflections from (10.0) planes were marked in the pattern. The growth direction of the nanotube coincides with the crystallographic *b*-direction (020) as indicated by a pink arrow and small green circles. It is evident from the TEM images and ED patterns that the nanotubes are single crystalline, and the MLC layers are grown periodically as superstructures. The atomic resolution (S)TEM image in Figure 2e shows the atomic arrangement in the rocksalt and trigonal prismatic lattices of SmS and TaS_2_, respectively. The structure constitutes a purely (*O–T*) arrangement related to the stage-1 misfit structure. The nanotubes produced at this temperature (825 °C) did not show the (*O–T–T*) arrangement related to stage-2 misfit structures (see Figure 1a). Figure 2f displays an FIB cut lamella of the (SmS)_1.19_TaS_2_ misfit nanotube and the corresponding HRSTEM image. The contrast in the STEM clearly shows the arrangement of misfit (*O*–*T*) and (*O–T*)′ (twisted by 30°) lattice with two folding vectors as revealed by the ED. This motif was found in different kinds of MLC nanotubes,^19^ suggesting that it is a rather common mechanism for stress relaxation during the growth of such nanotubes. Though TEM studies are advantageous for the detailed structural analysis of nanophases, they are limited to small sample volumes. Thus, it is quite tedious to study the thermodynamic and kinetic parameters in TEM and bring out a broad picture of the stability limits of the nanotubes. Hence, XRD techniques were employed to analyze large sample volumes and understand the stability limits of nanophases by analyzing suitable reflection (vide-infra).

### Diffraction (Reciprocal) Space of MLC Nanotubes

Starting from the pioneering work of E.J.W. Whittaker^20^ on the XRD studies of chrysotile, the peculiarities of the diffraction space of tubular structures have been revealed and extensively studied.^21−25^ First experimental works dedicated to the naturally occurring chrysotile and carbon nanotubes have shown that the diffraction space of nanotubes consists of diffuse curves, diffuse planes, and a limited number of sharp “nodes”.^23^ The set of parallel 2D crystal sheets wrapped into the cylinder gives rise to the usually sharp 00*l* nodes, represented as circles in reciprocal space. These nodes transform into sharp reflections in the 1D powder diffraction pattern. The distance between these peaks corresponds to the interlayer distance in the nanotube. The reciprocal space of the *hk*0 reflections associated with the parallel sheets tangential to the cylinder is more complicated^22,24,26,27^ and depends on the ordering between successive cylinders/turns: it can be either diffuse curves or planes. These types of reflections are seen as elongated streaks on the diffraction pattern if taken from individual nanotubes (as shown in Figure 2d).^21−23,28^ On the other hand, these *hk*0 are seen as highly asymmetric reflections with a long tail on the 1D diffraction pattern obtained from a nanotube powder.^29−31^ There is a translation disorder between adjacent layers in the nanotubes due to the differences in the circumference (number of atoms) between the layers. Owing to this stacking disorder, no sharp *hkl* reflections (with *l* ≠ 0, namely *h*0*l* and 0*kl*) occur,^21−23^ and they appear just as a reinforcement of *hk*0 diffraction peaks and sometimes as sharp peaks in polygonized nanotubes.^32^ Based on the analyses from previous studies and the present work, the conditions for reflection distinguish the nanotubes from the platelets and bulk particles, which are tabulated in Table 1.

**Table 1:** Reflection Conditions for the Most Common Phases in the Powder Containing Bulk, Single-layer 2D Materials, and Their Nanotubes^a^

reflections	bulk particles/Multilayered flakes (platelets)	nanotubes	single layers
00*l*	+	+	–
*hk*0	+	+	+
*h*0*l*	+	– (absent)^b^	–
0*kl*	+	– (absent)^b^	–

a The *h*0*l* and 0*kl* reflections are absent for nanotubes.

b Obviously, this forbidden peak is not absolutely excluded for large diameter tubes, where the curvature is small.

Since these first observations were done on carbon or chrysotile nanotubes and there were no systematic comparative XRD studies on tubular MLC compounds, additional verification is needed. First, all of the samples of the temperature and time series of (SmS)_1.19_TaS_2_ and (LaS)_1.14_TaS_2_ were characterized in detail using the laboratory XRD technique. Due to the highly intense and preferred (00*l*) reflection of misfit structure and lack of/poor information on (0*kl*) and (*h*0*l*) intensities to analyze nanotubes and their stability limits, XRD measurements at Petra III synchrotron were carried out, further. The large 0.3 × 0.7 mm X-ray beam was used to get an accurate X-ray intensity of weak *h*0*l* reflections, and a submicrometer X-ray beam was used to separate the XRD signal of single nanotubes from bulk flakes. The great advantage of the submicron X-ray beams in comparison to electron diffraction is that the former has a much larger penetration depth and, therefore, even large particles can be studied.

### Conventional XRD Measurements

The XRD patterns of MLC powders (flakes+nanotubes) of (SmS)_1.19_TaS_2_ and (LaS)_1.14_TaS_2_ were collected first using a lab-based X-ray source in reflection geometry. The powder patterns for (SmS)_1.19_TaS_2_ for both temperature and time series are shown in Figures S6 and S7, respectively. The reflections match well with the reported phase of (SmS)_1.19_TaS_2_.^33^ The patterns consist of highly oriented strong (00*l*) reflections, and it was necessary to plot them in log intensity to see the Bragg reflection other than (00*l*), which are quite useful to deduce the information on abundance and stability.

### XRD Analysis of (SmS)_1.19_TaS_2_ Temperature and Time Series

The samples prepared in the temperature regime between 800 and 975 °C show highly preferred (00*l*) reflections with a periodicity of 11.14 Å/n (*c*/2) corresponding to (*O–T)* superstructure (stage 1) MLC (Figure S6). As per the above argument proven in the case of CNTs and INTs, the reflections from (0*kl*) planes such as (022), (024), and (026) are important information to distinguish the bulk/flakes from the nanotubes. The 0*kl* reflections of the present MLCs appearing between 2θ 30° and 40° (indicated in cyan color) are visible, but their intensities are very poor and are highly asymmetric. Hence, it is quite challenging to distinguish (filter) the information of nanotubes and flakes. Nevertheless, the intensity of 0*kl* reflections continues to grow with growth temperature, especially the (026) planes, indicating a higher population of the flakes/bulk particles, which observation is consistent with SEM analysis. The XRD pattern of samples prepared above 950 °C constitutes an additional series of 00*l* reflection with a periodicity of 17.4 Å/n (*c*/2), which corresponds to (*O–T–T)* superstructure of stage 2 MLC (refer Figure 1a). The stoichiometric ratios of all the precursor elements used for the synthesis were kept strictly the same for all the compounds prepared in this study. The additional 00*l* reflections of the (*O–T–T*) phase indicate the stoichiometric deviation and refer to the leaching out of the rocksalt SmS lattice from the pristine (*O–T*) phase. So, to preserve the Stage 1 structure of the MLC nanotubes, it would be better to choose temperatures below 950 °C, since the properties that are influenced by stacking and charge transfer of (*O–T*) and (*O–T–T*) differ largely.

**Figure 3:**
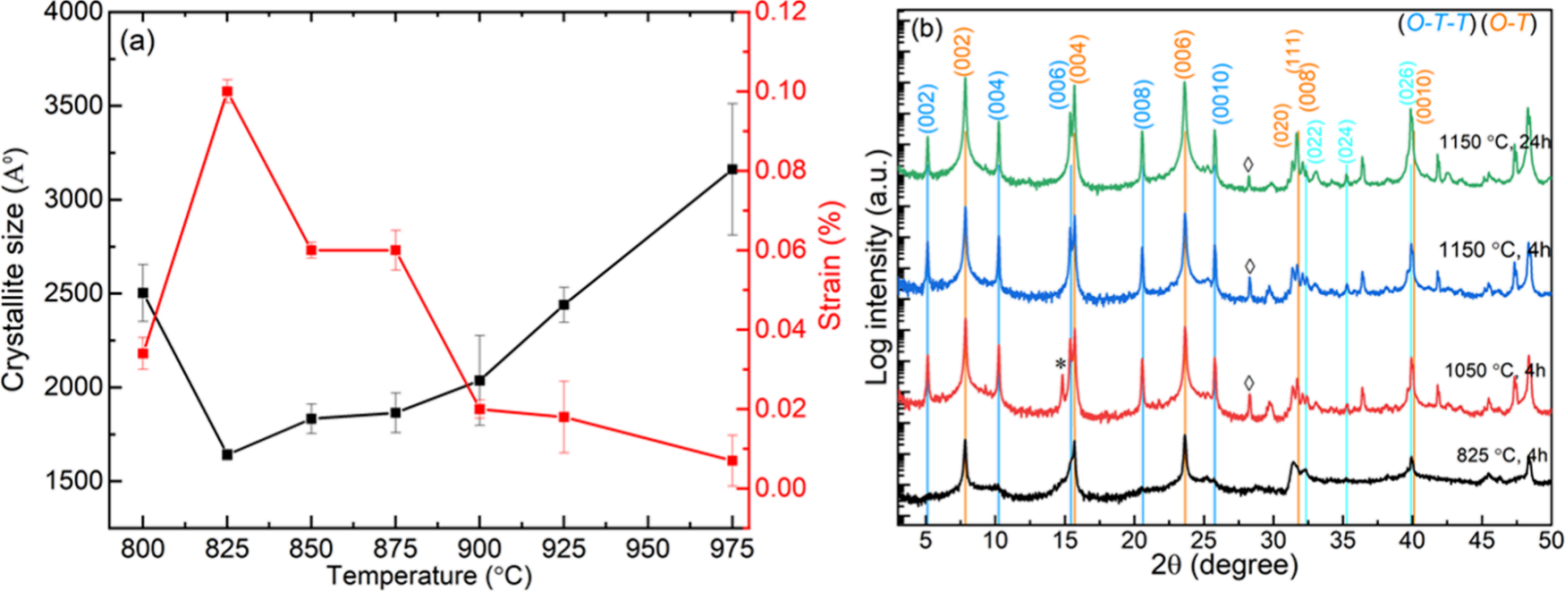
XRD analysis of (SmS)_1.19_TaS_2_ samples. (a) Analysis of crystallite size and the strain of samples prepared at different temperatures. Williamson–Hall analysis was done by considering the peak widths of selected 00*l* reflections from (*O–T*) stacking. (b) Powder XRD patterns of the (SmS)_1.19_TaS_2_ sample (flakes + nanotubes) prepared at 825 °C for 4h in comparison with the annealed samples above 1000 °C. The 00*l* Bragg reflections associated with the (*O–T*) and (*O–T–T*) superstructures with *c*-axis periodicity 11.2 and 17.2 Å, respectively, are marked with orange and blue solid lines, respectively. Please refer to the SI Figures S6 and S7 for the XRD analysis of products obtained for the (reaction) time and temperature series. The peaks marked in diamond symbol in the range 28° in high temperatures and time correspond to cubic(I) Sm_2_O_3_ (PDF 04-004-8958)/orthorhombic Sm_2_Ta_3_S_2_O_8_ phases (PDF 04-011-1697).

WS_2_ nanotubes display higher strain and smaller crystallite size compared with flakes and bulk crystals. The analysis of these parameters would give insights into the abundance and stability of the nanostructures in MLC powders. The strain and the crystallite size of the samples prepared at various temperatures were calculated using Williamson–Hall (W–H) analysis, and the results were summarized in Figure 3a. The smallest crystallite size and highest strain are observed for the sample grown at 825 °C, and it indicates the presence of a high number of nanotubes, compared to the rest of the samples. These results correlate well with the SEM analysis where the highest abundance of the nanotubes (strain) with the smallest diameters (crystallite size) was observed for the samples grown at 825 °C (Figure 2b). Although initially strain relaxation is believed to be a predominant driving force for the folding of the MLC layers, the final multiwall nanotubes may reveal higher strain than the flakes owing to the strong driving force dictated at small diameters for the seaming of the dangling bonds of the rim atoms. Indeed, the strain drastically decreases above 875 °C and relaxes to a minimum value, and the crystallite size grows bigger and bigger, signifying the growth of larger platelets and microtubules, which again well aligns with the SEM data (Figures 2b and S3). Unlike SEM and TEM, the above analysis allows us to deduce the macroscopic picture of the preferred thermodynamic and kinetic regimes for the growth of nanotubes in copious amounts.

The powder XRD patterns of the (SmS)_1.19_TaS_2_ specimen with up to 4 h annealing times show strong reflections of (*O*–*T*) superstructure (Figure S7) similar to the temperature series. The sample grown for a longer duration >6 h shows additional 00*l* reflections corresponding *to* the (*O–T–T*) superstructure of (SmS)_1.19_(TaS_2_)_2_, indicating the transformation of a portion of the sample from (*O–T*) to (*O–T–T*). The mechanism of conversion of (*O*–*T*) (<6h) to (*O*–*T*–*T*) would be due to the leaching of the SmS layer from the pristine (*O–T*) lattice. The absence of (*O–T–T*) reflection in shorter growth duration affirms this notion (see also the discussion below). In addition, the peak at 28° assigned to cubic Sm_2_O_3_ emerges at 8 h corresponding with the growth of the (*O–T–T*) phase. This finding indicates that SmS leached from the (*O–T*) superstructure and oxidized into Sm_2_O_3_ by reacting with the unwieldy residual oxygen present in the quartz ampule.

To understand the thermodynamic stability of the MLC nanotubes/flakes, the samples prepared at 825 °C, 4h (Figure 2a) were further subjected to an annealing process above 1000 °C for several hours in evacuated quartz ampules. Figure S8 shows a series of SEM images of annealed samples; the thinner nanotubes and flakes (Figure 2a) were converted into larger microtubules and thicker platelets with several μm lateral dimensions. Both annealing temperature and time have a great influence on the growth of microcrystallites, which is evident from the samples annealed at 1150 °C, 4 h and 1150 °C, 24 h (Figure S8c–f). The EDS (SEM) analysis shows stoichiometric deviation from the (*O–T*) phase and excess TaS_2_ in the microtubules and platelets indicating the presence of (*O–T–T*) superstructure. Few samples from the high-temperature synthesis were analyzed by TEM. Figures S9 and S10 show the TEM images of the annealed sample in comparison with pristine nanotubes. The surface of the annealed sample shows discontinuous and also modulated structure which is entirely different from the atomically smooth inner layers of the studied nanotubes. This may be due to either smaller crystallites coming closer and coarsening into microcrystallites or due to the leaching of SmS layers. Since these crystallites are quite big and nontransparent for electrons, it poses difficulty in analyzing them in greater detail.

To further confirm the degradation mechanism and understand the fate of the inner layers and the hollow core of the nanotubes, the annealed tubes were cut through FIB, and thin cross-sectional lamellae were prepared for TEM analysis. The HAADF-STEM image of one such lamella (from the sample annealed at 1150 °C, 4 h) is shown in Figure S11. Owing to Ostwald ripening at the elevated temperature annealing, the tube (scroll) grew to a diameter of 500 nm, making it an intermediate case between the original nanotube and bulk MLC flakes. The core of the tube (scroll) shows modulation similar to the surface of the nanotubes (Figure S11b). The undulations of the layers are also visible for the surfaces near the crack in Figure S11c. More importantly, the analysis reveals that SmS layers squeeze out (deintercalate) from the *O–T* superlattice and form *O–T–T* layers. Several *O–T–T* layers can be observed throughout the cross-section of the tube and are marked by cyan lines (Figure S11d). In the upper defect marked by two cyan lines, the (*O–T–T*) arrangement has already been formed on the left, while the original (*O–T*) stacking occurs on the right side. This defect clearly reveals the deintercalation of the SmS layer from the MLC structure. These results are in line with the XRD analysis (vide infra). The MLC flakes from the sample also show a similar mechanism of degradation forming (*O–T–T*) layers (Figure S12). Finally, the nanotubes prepared at 825 °C were further annealed at 1200 °C for 4 days. All the misfit nanotubes were converted into 2D flakes (Figure S13). The XRD patterns confirm that the crystals at the hot zone comprise TaS_2_ (major phase) and misfit (SmS)_1.19_TaS_2_ minor phase, whereas the crystals at the cold edge were found to be purely TaS_2_ (Figure S12c). This observation confirms that at substantially higher temperatures and long annealing hours, the misfit nanotubes completely disintegrate into binary phases.

Further, the annealed samples (microcrystallites) were analyzed by XRD and are shown in comparison with nanotubes grown at 825 °C in Figure 3b. The predominant conversion of the (*O–T*) phase to the (*O–T–T*) phase is evident upon annealing. The 0*kl* reflection, i.e., the 026 peak, which is a marker for bulk platelets (Table 1), grows with annealing temperature and time indicating the successive conversion of nanotubes into platelets and microcrystallites. Though it is difficult to quantify the platelets due to the overlapping of the peaks and resolution limit in the measurements, the qualitative agreement can be drawn from the XRD and microscopy analyses for the conversion of nanotubes into microcrystallites. The appearance of a new peak at 28° corresponding to cubic Sm_2_O_3_ further confirms the leaching of SmS and its subsequent oxidation. Annealing above 1150 °C requires a much more sophisticated reactor design and annealing system since quartz ampule fails to withstand higher temperatures. The results from lab-based XRD, microscopy (SEM and TEM), and EDS analysis were informative to understanding the structure, composition, and stability (to some extent) of the nanotubes, but due to the narrow field of view (microscopy) and limited resolution (XRD), the information obtained is qualitative. Hence, it was difficult to quantify the structural transformation from (*O–T*) to (*O–T–T*) and more importantly the kinetic and thermodynamic stabilities for the conversion into microcrystallites. Synchrotron-based X-ray measurements were employed to get quantitative information on the nanotube abundance and insights into the thermodynamic and kinetic stability of nanophases.

### Single Nanotube and Powder Diffraction with Synchrotron Radiation

**Figure 4:**
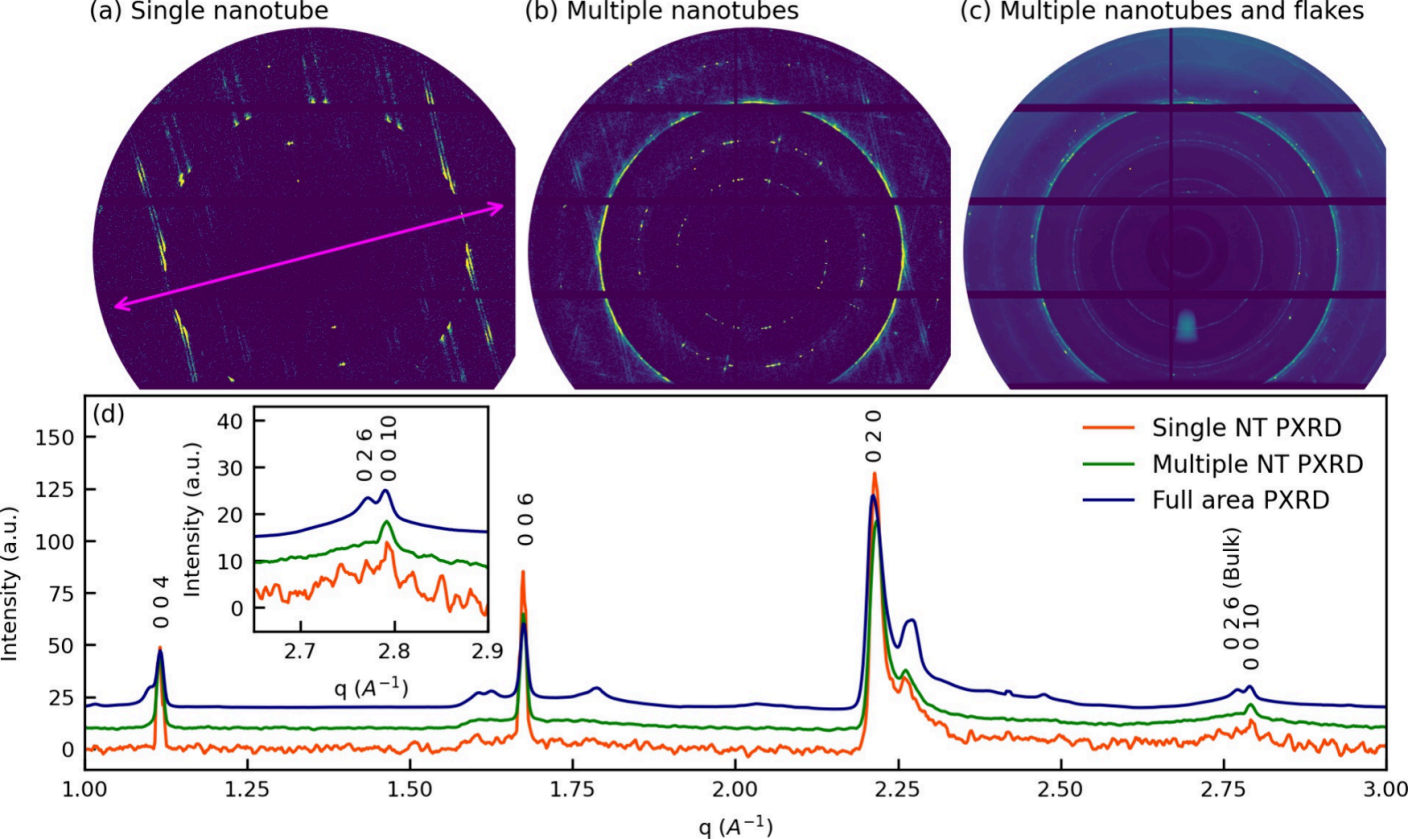
XRD patterns obtained with sub-μm X-ray synchrotron beam from (SmS)_1.19_TaS_2_ prepared at 825 °C. (a) XRD pattern from a single nanotube; the nanotube axis is marked with pink double arrow; (b) integrated XRD pattern collected from many different nanotubes; (c) integrated XRD pattern from the entire (SmS)_1.19_TaS_2_ sample containing both nanotubes and flakes. (d) Azimuthally integrated (a), (b), and (c) patterns imitating filtered 1D X-ray diffractograms from single nanotube, pure nanotube powder, and full sample containing nanotubes and MLC flakes.

First, synchrotron X-ray studies with a sub-μm X-ray beam were performed to distinguish the diffraction features of nanotubes and platelets that were spatially separated on the TEM grid. During the experiment, the TEM grid with the (SmS)_1.19_TaS_2_ sample (grown at 825 °C for 4 h) was scanned with a sub-μm X-ray beam, and the XRD patterns were collected. Such an approach allows one to filter the diffraction images that belong purely to the nanotubes from those that also contain the signal from the MLC platelets. Figure 4a displays the XRD pattern of a single nanotube; the XRD pattern from many different nanotubes summed up together to get better statistics (Figure 4b) and integrated XRD pattern from the full area of the sample (Figure 4c). Figure 4d represents azimuthally integrated 2D patterns (Figure 4a–c, correspondingly) imitating the powder XRD from the single nanotube, a pure nanotube powder, and the whole sample containing both nanotubes and platelets. From Figure 4d, it is clearly seen that the nanotube shows only strong 00*l* reflections and those of the highly asymmetric *hk*0 (namely, 020) planes. The sharp 026 reflection (at *q* = 2.78 A^–1^) occurs only for data collected from the full area of the sample, indicating that it belongs to the MLC platelets.

Thus, like WS_2_ or chrysotile nanotubes, MLC nanotubes have several unique diffraction features, that distinguish them from the conventional 3D crystals and single 2D layers, that are based on the same structural units/layers (WS_2_ nanotubes vs tungstenite (2H-WS_2_), carbon nanotubes vs graphite etc.). These features are summarized in Table 1. Contrary to the multilayered MLC nanotubes and in the absence of repeating planes along the *c*-axis, single 2D MLC layers do not display the 00*l* reflections. These diffraction features can be used to get specific information about the nanotubes in the raw powder containing both flakes and tubules. The width of the 00*l* reflections can be used as an estimate of the nanotube thickness. Furthermore, the 00*l*/0*kl* reflections ratio can be used as a semiquantitative indicator for nanotubes abundance (to be more precise MLC NTs/MLC flakes ratio).

### Temperature Series of (SmS)_1.19_TaS_2_ Nanotubes

**Figure 5:**
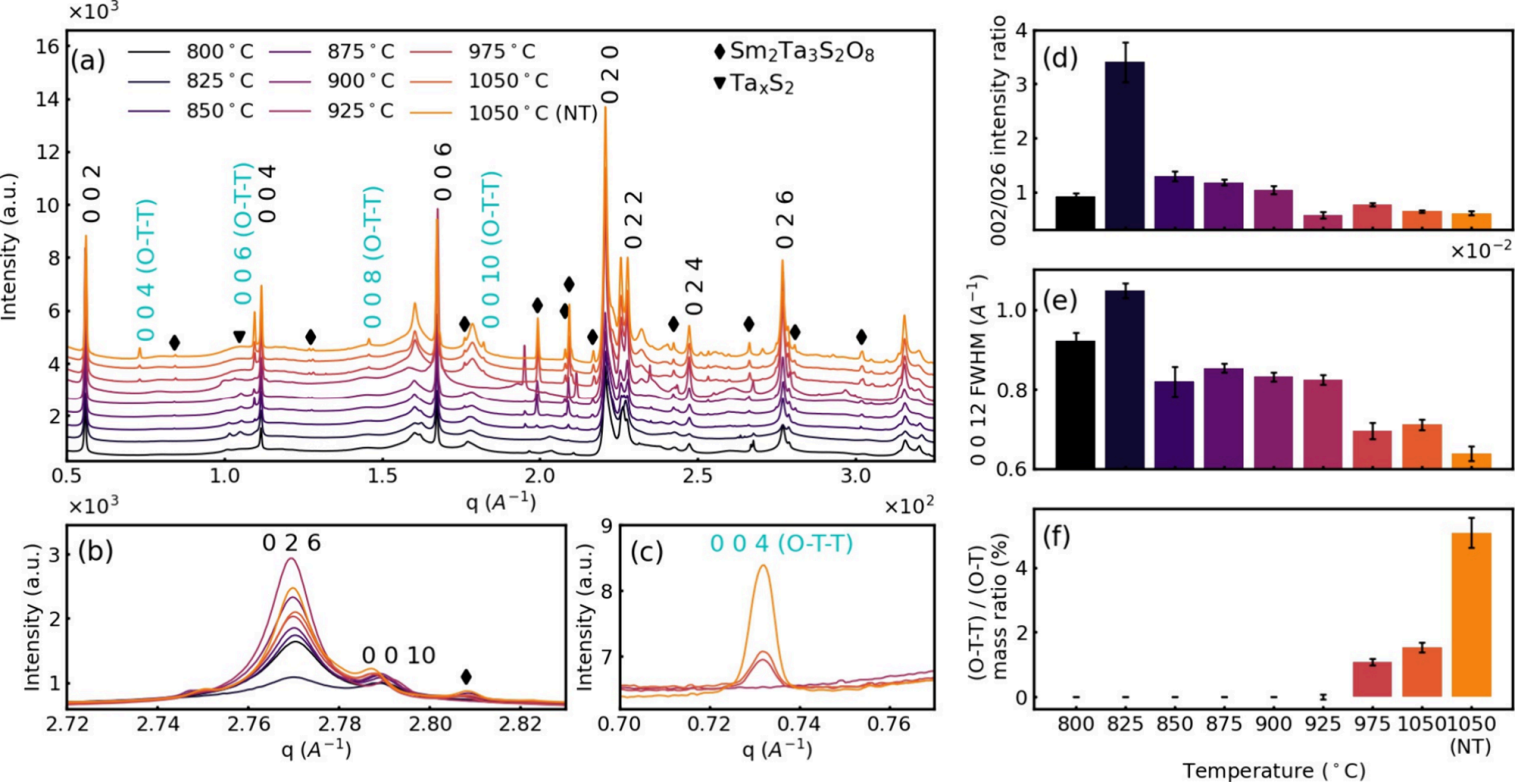
X-ray diffractograms of the temperature series (SmS)_1.19_TaS_2_ samples: wide-range diffractograms (a), enlarged ranges of the (*O–T*) 026 (b), and of *(O–T–T)* 004 (c) reflections from of (SmS)_1.19_TaS_2_. The stage 1 (*O–T*) and stage 2 (*O–T–T*) superstructure reflections were marked in black and cyan color. The reflections from the impurity of Ta_*x*_S_2_ are indicated with symbol ▼ and the oxidized SmTaSO_8_/Sm_2_O_3_ by ◆. Intensity ratios of 002/026 reflections (d), fwhm of the 0012 reflection (e), and (*O–T–T*)/(*O–T*) mass ratios (f), calculated from 002 (*O–T*) and 004 (*O–T–T*) reflections as a function of temperature.

XRD analysis was carried out for all of the tabulated samples of the temperature and time series (Table S1). The patterns are consistent with the previous measurements and match well with the reported phase of the (SmS)_1.19_TaS_2_ stage-1 (*O*–*T*) superstructure. Unlike lab-based XRD data shown before, the synchrotron measurements pick up well the 0*kl* reflections of the (*O–T*) superstructure, i.e., 022, 024, and 026, which appear only in the flakes (absent in nanotubes). The 026 reflection is the most intense among the 0*kl*, and it is well resolved from the 0010 reflection. Hence, it is further used for the present analysis (Figure 5b). The 002/026 reflection ratio, which serves as an indicator for the nanotube content, decreases with the growth temperature (Figure 5d), showing that the abundance of the nanotubes is reduced with increasing temperature, which is in line with the SEM observations. One can note two steps on this plot (Figure 5d): the highest nanotube abundance is found at 825 °C, then it decreases significantly at 850 °C. The nanotube content is almost constant in the 850–925 °C temperature range and then it drops down again at 975 °C. Surprisingly, the full width at half-maximum (fwhm) of the 0012 reflection (Figure 5e) follows the 002/026 ratio trend and is high for the sample prepared at 825 °C which comprises a high abundance of the nanotubes. Indeed, the fwhm decreases with the annealing temperature, and the peak becomes narrower, indicating coarsening of the crystalline domains due to the disappearance of the nanotubes and the formation of the corresponding MLC flakes.

With increasing temperature above 950 °C, the MLC with (*O–T–T*) superstructure periodicity, i.e., (SmS)_1.19_(TaS_2_)_2_ starts to grow (Figure 5c), with the highest intensity of the 004 (*O–T–T*) reflection observed for the annealed sample at 1050 °C, indicating the structural transformation. The formation of the (*O–T–T*) structure at 975 °C clearly explains the two-step behavior of Figure 5d,e plots: (*O–T*) nanotube abundance is going down again at 975 °C because the (*O–T*) nanotubes start to transform into the (*O–T–T*) structure. The 1050 °C (NT) sample corresponding to the sample synthesized at 825 °C and annealed at 1050 °C has a higher intensity of the 002 (*O–T–T*) reflection in comparison with the sample prepared directly at 1050 °C. Therefore, it seems that the (*O–T*) nanotubes, which are formed in large yields at the 825 °C sample would act as a “template” seeding the (*O–T–T*) SmS-(TaS_2_)_2_ growth in the 1050 (NT) sample. The significant increase in (*O–T–T*)/(*O–T*) in annealed samples indicates that high temperature annealing facilitates the conversion of the (*O–T*) phase into (*O–T–T*) phase due to the inferior thermodynamic stability of the former at elevated temperatures. Semiquantitive phase analysis (Figure 5f) based on relative intensities of (*O–T–T*) and (*O–T*) reflections shows that the (*O–T–T*) mass fraction is below 6% of (*O–T*) nanotubes. From this analysis, one can conclude that increasing the temperature used for the synthesis of the MLC leads to the gradual conversion of the nanotubes into MLC flakes and, simultaneously also, to the conversion of the (*O–T*) phase into (*O–T–T*) one.

### Time Series (SmS)_1.19_TaS_2_ Nanotubes

**Figure 6:**
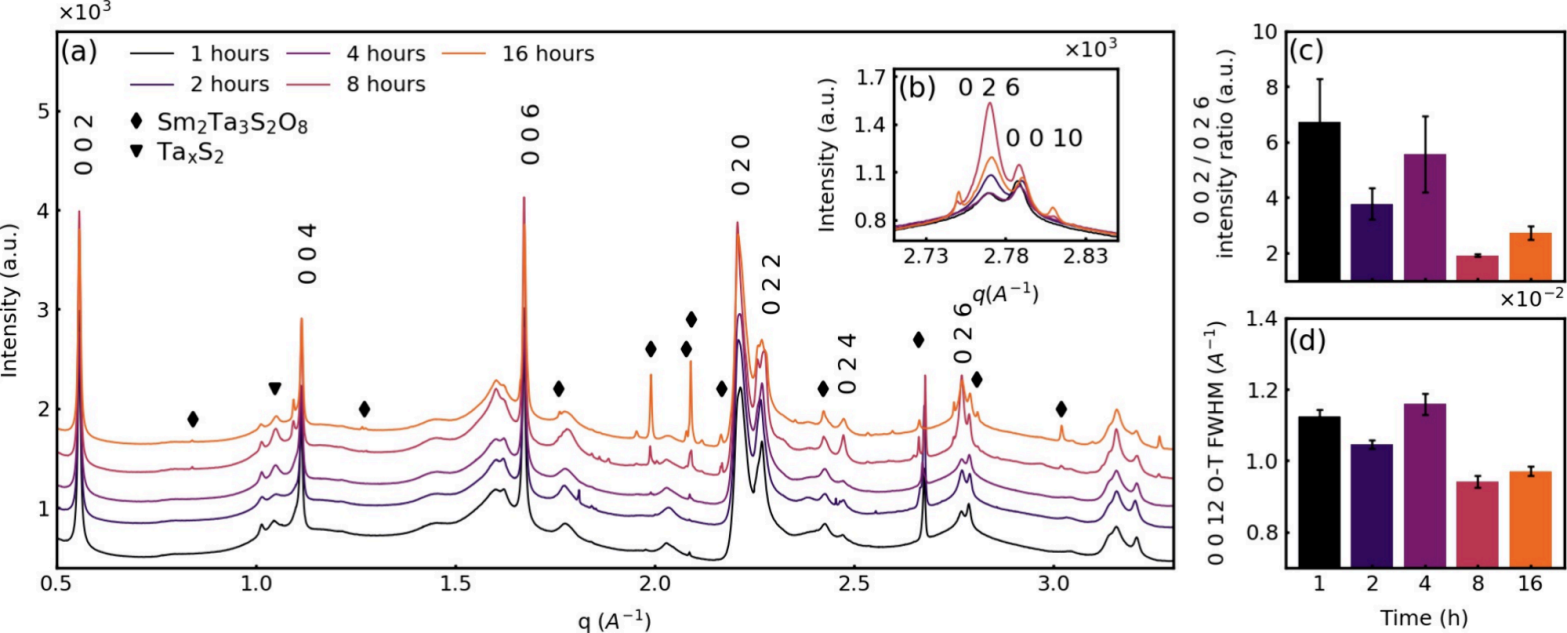
(a) X-ray diffractograms of (SmS)_1.19_TaS_2_ samples obtained at 825 °C with varying reaction time (1–16 h) at different *q* ranges. The plots were shifted vertically for clarity. Inset (b) shows exploded view of the (026) and (0010) reflections; intensity ratio of the 002/026 XRD reflection (c) and fwhm of the 0012 reflection (d) as a function of annealing time.

It is also clearly seen that the intensity of the 026 reflection, which corresponds to the (SmS)_1.19_TaS_2_ bulk particles (platelets) alone, grows with time as well (Figure 6a,b). As stated above, the 002 reflection corresponds to both the (SmS)_1.19_TaS_2_ nanotubes and bulk particles, while the 026 corresponds to bulk particles only (Table 1). Therefore, the 002/026 reflection intensity ratio can serve as a semiquantitative indicator for the nanotube content in the sample. Indeed, it is clearly seen that the 002/026 ratio (relative nanotube content) decreases with the heating time (Figure 6c). Surprisingly, a similar trend is also seen in the full width at half-maximum (fwhm) of the 0012 reflection. Indeed, the fwhm also decreases with heating time. Decreasing fwhm of the 0012 peak along the time series (Figure 6d) is attributed to the growth of the crystallite size and, probably, to a reduction in the defect density, which again is compatible with diminishing nanotube concentration with annealing time and increasing the amount of large (SmS)_1.19_TaS_2_ flakes. It was found that trace amounts of both tantalum sulfides Ta_1.2_S_2_ and Ta_1.08_S_2_ were present in all the samples, and this observation was not clear from lab-based XRD studies. The residual binary TaS_2_ phases can possibly be ascribed to minor deviations from stoichiometric composition in weighing of the precursors. The intensity of these phases increased after prolonged annealing. The intensity of the Sm_2_Ta_3_S_2_O_8_ (ICDD PDF-2 01-083-0999) phases starts to grow prominently at 875 °C (Figure 5a). The intensity of Sm_2_Ta_3_S_2_O_8_ starts to grow also after 8 h of heating at 825 °C (Figure 6a). These measurements suggest that the pure (SmS)_1.19_TaS_2_ misfit phase starts to degrade by reacting with residual water and oxygen in the sealed ampule. As careful as possible, the quartz glass always contains a non-negligible water residues, which diffuse out slowly at elevated temperatures and react with the MLC phase. Another possibility is a high-temperature reaction between the reactants and the silica of the tube wall, which produces these oxides. This analysis indicates that an additional mechanism for the high-temperature degradation of the MLC nanotubes is the reaction of the (*O–T*) nanotubes (and flakes) with water or silica to produce samarium–tantalum oxides.

### General Discussion

The first nanotubular phases studied were the “chlorites”, i.e., layered alumino (magnesio) silicates.^34^ By analyzing Laue photographs, Pauling noticed that some of the layered chlorites, like halloysite (hydrated form of kaolinite Al_2_Si_2_O_5_(OH)_4_·2H_2_O) and chrysotile Mg_3_Si_2_O_5_(OH)_4_ consist of two subunits, i.e., a slab of silica tetrahedra and another slab of alumina (magnesia) octahedra connected via vicinal oxygen atoms. The two subunits are incommensurate along the *a*-axis (asymmetric layered structure), and consequently, he concluded that such asymmetric layered structures are bound to relax by folding.^35^ Surprisingly, model calculations revealed that single or few-layer chrysotile^36^ and imogolite^37^ nanotubes are thermodynamically stable; i.e., they exhibit an energy minimum below the energy of the infinite flat layer.^38,39^ These observations may explain their natural occurrence over geological time scales on the earth's surface.

On the other hand, the driving force for the formation of nanotubes from layered compounds, like MoS_2_, which are symmetric is totally different and is believed to stem from the abundance of dangling bonds of the rim atoms (large ratio of rim/bulk atoms at the nanoscale). Model calculations^4,40^ show that for layered compounds with symmetric structure, like MoS_2_, the elastic energy of folding is always positive and larger than the energy of the infinite (flat) layer. However, the strain energy of the nanotubes is smaller than the energy of dangling bonds of the flat nanoribbons in a certain size range (approximately 5–100 nm).^7,8^ Hence, unlike chlorites, the nanotubes from symmetric TMDCs are generally metastable.

In view of this discussion, the question arises whether MLC nanotubes are stable or a metastable structure. The most straightforward way to address this issue would be through an ab initio or related calculation. However, given the size of the unit cell (of the MLC approximant), this calculation is probably beyond the reach of the presently available computation power. Furthermore, ab initio calculations are generally limited to zero Kelvin and cannot directly address the high-temperature stability of nanotubes.

The high-temperature stability of ternary compounds with a layered structure did not receive much attention in the literature, let alone their (metastable) nanostructures. Nonetheless, the thermal stability of alkali-metal-intercalated binary compounds was studied, mostly via differential scanning calorimetry. Early on, Wittingham studied the temperature (up to 100 °C) dependence of lithium intercalation in TiS_2_.^41^ Expectedly, he found that the entropy increased with temperature very mildly, leading to reduced stability of the intercalated phase compared to a pure TiS_2_ and the metal phases. In another study, the phase equilibrium of copper intercalated in the layered compound ZrTe_2_ was investigated as a function of long annealing in different temperatures (250–1000 °C).^42^ It was found that the amount of copper intercalated phase in ZrTe_2_ diminished with increasing temperature. Exfoliated (via lithium intercalation) and restacked MoS_2_ was found to consist of mixed 1T and 2H polytypes. Intercalating the restacked MoS_2_ with Co(OH)_2_ and cobaltocenium ions produced a negatively charged host.^43^ Upon heating, the guest atoms left the MoS_2_ host, which gradually transformed back into a stable 2H polytype. These works and others show that owing to the free entropy gain, prolonged heating of ternary compounds at elevated temperatures induces decomposition into their constituent binary compounds and pure elements.

As discussed in a previous study, substituting sulfur with selenium in the anion site of the MLC, LaS*–*TaS_2_ nanotubes (and flakes), led to a gradual structural transformation of the (*O–T*) phase into (*O–T–T*) upon excess selenium.^17^ This transformation is attributed to the fact that the sulfur atoms bind exclusively to the lanthanum atoms. This chemical selectivity is surprising, given the high temperature of the reaction (>825 °C), which is conducive to a randomly distributed sulfur and selenium atoms and thereby gaining high entropy of mixing. Since the stability of rare-earth-based misfit compounds is associated directly with the charge transfer from the *Ln*-atom to the tantalum atom, it is clear that the periodicity (*O–T*) is more stable than the (*O–T–T*) one, where the charge transferred from the rare-earth atom is shared between two neighboring tantalum atoms. The absence of a stable (*O–T–T–T*) phase in the product strongly suggests that the middle (*T*) layer is unstable owing to the lack of a direct interface with the (*O*) layer, which is a requisite for an effective charge transfer. The present study shows that the high-temperature stability of nanotubes (and flakes) of the misfit compounds (LnS)_1+*y*_(TaS_2_)_*m*_ follows the same path, i.e., gradual transformation of the most stable (*O–T*) phase into the less stable (*O–T–T*) phase (and leaching out of the *O* phase), which decomposes under excessive annealing temperature and time (and also annealing the powder at a temperature higher than the one used for the synthesis). Although the products of the decomposition were not studied in great detail, the binary SmS, TaS_2_, and their corresponding mixed phase oxides are the common byproduct of the excessive annealing (as shown by XRD).

The thermal degradation of fullerene-like (IF) NPs of WS_2_, MoS_2_, and NbS_2_ was investigated and compared to the corresponding micron-sized platelets in both inert and ambient atmospheres.^7^ As anticipated, the IF NPs were found to be somewhat less stable against high-temperature decomposition in inert and oxidizing atmospheres compared with the bulk platelets. These experiments could not resolve any difference between the chemical decomposition pathways of the NPs and the flakes of the same compound. Therefore, in contrast to chrysotile, halloysite, and imogolite, the nanotubes of such (symmetric) layered compounds are metastable and are globally less stable than the bulk crystallites (flakes). Similarly, this study, which has used entirely different research methodologies, could not distinguish between the decomposition routes of the MLC nanotubes and flakes. That does not mean that such differences do not exist, but they require a different set of experimental and theoretical tools, which are beyond the scope of the present study and could be a topic of a future study.

While these observations were made for both the metastable MLC nanotubular phase and the bulk flakes, the latter seemed to be the more stable of the two, as indicated by the large body of measurements brought up in this work. The decomposition of misfit nanotubes (and flakes) at elevated temperatures is not unprecedented. However, the (*O–T*) into (*O–T–T*) transformation and subsequent decomposition are unique to the MLC phases warranting further studies.

## Conclusions

Generally, nanostructures and NPs are metastable, and their degradation mechanism at elevated temperatures has been briefly discussed in the literature, which is the main topic of the present research. The optimized growth conditions for the nanotubes of two model compounds from the lanthanide-based MLC family were thoroughly investigated using a combination of electron microscopy, lab-based, and synchrotron X-ray measurements. Copious amounts of nanotubes from these compounds were grown at 825 and 875 °C, respectively, and the accounted procedure is highly reproducible. Of the halide series, chloride salt was found to be the most effective transport agent in the reaction. The high-temperature transformation of the (*O–T*) phase, which is stable at low temperatures into the (*O–T–T*) phase and MLC flakes, and subsequently into the corresponding binary SmS_*x*_ and TaS_2_ phases and residual oxides, was investigated. The annealing temperature of quartz ampules (1150–1200 °C) limited the current investigation of this study. Therefore, the complete degradation mechanism of the (*O–T*) phase into the (*O*) and (*T*) phases going through the (*O–T–T*) phase, and its complete decomposition could not be fully studied here. Vacuum sealing the MLC nanotubes in thicker tantalum tubes and annealing at temperatures (>1400 °C) using MoSi_2_/graphite furnaces would help to understand the degradation of MLC flakes/crystals but this is beyond the scope of the present study, which was focused on the fate of the MLC nanotubes. The findings of this work partially address the kinetic and thermodynamic stability lifespans of misfit nanotubes and the general issue of metastability of nanostructures. The scrutinized time and temperature parameters would allow researchers to successfully grow these nanotubes and investigate their enticing physical properties, such as superconductivity, magnetism, topological materials for quantum technologies, and other many-body physical properties.

## Experimental Section

### Synthesis of (LaS)_1.14_TaS_2_ and (SmS)_1.19_TaS_2_ Misfit Nanotubes

In the present study, (LaS)_1.14_TaS_2_ and (SmS)_1.19_TaS_2_ misfit nanotubes are used as model systems for the study of the growth kinetics and stability with respect to annealing temperature and time. The nanotubes were synthesized using a well-established CVT protocol in evacuated quartz ampules^13,44^ at different reaction temperatures and times. The reactants and the products were handled under an inert atmosphere provided by a glovebox in order to prevent their oxidation. A stoichiometric amount of La/Sm (STREM, 99.9% REO), Ta (Alfa Aesar 99.9%), and S (Sigma-Aldrich 99.98%) was mixed in an agate mortar in the 1.14:1:3.14 (La:Ta:S) molar proportion. A small amount (3 mg) of TaCl_5_ (Sigma-Aldrich 99.99%) was used as a catalyst, which upon decomposition produces chlorine acting as a transport agent. Owing to the small amount of weighted materials, minor deviations from the stoichiometric composition of the MLC phases were put in the ampules. This resulted in occasional minor amounts of TaS_2_ (see for example the XRD in Figure 5). The quartz ampules were sealed under vacuum (<1 × 10^–5^ Torr) and transferred to a preheated two-zone vertical furnace for the annealing process. The annealing was performed in two steps using two opposite gradients of the temperature under constant monitoring. In the first step, the ampules were submitted to a thermal gradient of 400 °C at the bottom edge and 800 °C at the upper edge. After 1 h, the ampules were moved inside the bore of the furnace and exposed to various temperatures and time intervals to study the growth, kinetics, and thermodynamics. After the reaction, the ampules were naturally withdrawn from the furnace and allowed to cool to room temperature. For each La and Sm atom, various reactions were carried out under different conditions (see Table S1 for sample details) to reveal the right conditions for the nanotubes' growth. Reactions with various transport agents (Cl, Br, and I) were also carried out under optimized temperature and time conditions to identify the right transport agent for the nanotubes’ formation. To learn the effect of quenching on nanotube formation, a few reactions were carried out and were subsequently quenched at faster rates using water and liquid nitrogen. The mass transport was negligible in all the products including the slowly cooled sample, and the product was accumulated in the high-temperature edge of the ampule. The prepared product was stored in a glovebox and used for further analysis. The structural analyses were done using lab-based XRD, scanning electron microscopy, and transmission electron microscopy, refer to SI text.

### Synchrotron XRD Studies (Sub-μm Beam and Powder XRD)

Experiments were performed at the DESY *P*23 “in situ and X-ray imaging beamline”. The experimental setup for powder XRD consists of entrance slits, a first intensity monitor, and X-spectrum Lambda 750 K Si (pixel size 55 μm, 1536 × 512 pixels) 2D diffraction detector (Figure S14). Liquid N_2_-cooled double crystal monochromator (Si 111) was used to establish 9.68 keV X-ray energy for powder XRD and 10.69 keV for single nanotube XRD with focused X-ray beam. X-ray mirrors with a B_4_C coating were used for harmonic rejection.

In order to reduce the influence of preferable orientation in the powder diffraction, the MLC powders were mixed with cellulose binder (ratio 1:3) and pressed with a hydraulic press (2 ton) to produce 10 mm pellets suitable for analysis in transmission geometry. To avoid possible oxidation of the samples, mixing and handling of the powders were performed inside the Ar-filled glovebox (c(H_2_O) and c(O_2_) < 1 ppm). In order to avoid atmospheric contact during the measurements, the sample pellets were placed before the analysis between two Kapton foils (thickness: 50 μm) inside a special sample holder forming a closed volume. The sample pellet was mounted on the OWIS DRTM 40 rotary stage and rotated with a speed of 180°/s during the measurement. LaB_6_ powder (NIST 660c) was used as a standard for the calibration of the detector distance.

For single nanotube XRD experiments, nine Beryllium compound refractive lenses were used to focus the X-ray beam down to 0.8 × 3 μm size. The sample was prepared using the same protocol as for the case of the TEM (drop casted on SiN membrane window) studies and kept in a vacuum during measurements. During the experiment, the SiN membrane was scanned with a sub-μm X-ray beam with a step of 2 μm, and XRD patterns in transmission were collected at each point. The DECTRIS PILATUS 1 M Si detector was used for single nanotube XRD.

### DFT Simulations

The energetics of the different (*O–T*) compositions were calculated in the framework of DFT. Because of the large size of the nanotubes, at the atomic scale, the nanotubes can be approximated by their 2D configuration, discarding the curvature effects.^45^ We have thus modeled all structures as 2D layers (Figure 1a). The geometry optimizations (unit cell size and atomic positions) were carried out by the Vienna Ab-Initio Simulation Package (VASP)^46,47^ in the generalized gradient approximation using the Perdew–Burke–Ernzerhof (PBE) exchange-correlation functional.^48^ The method used to describe the interaction between core and valence electrons was the projected augmented wave pseudopotential.^49^ For the valence electrons, their wave functions were represented using a plane-wave basis set, which was chosen with a kinetic energy cutoff of 500 eV. A 5 × 1 × 5 k-point sampling was employed within the first Brillouin zone. Additionally, the DFT-D3 correction as per Grimme’s method was applied to accurately represent interlayer van der Waals interactions.^50^ The structures were relaxed until the residual energies and forces were below 10^–5^ eV and 10^–2^ eV/Å, respectively. Construction of the structures and analysis of the results were performed using the Atomistic Simulation Environment (ASE).^51^

An approximant in the context of MLC refers to a structure that approximates the mismatch in the lattice parameters between two different crystal layers. This effectively minimizes the lattice mismatch strain energy by introducing a larger unit cell that can accommodate both lattices with minimal distortion. The MX unit exhibits a distorted rocksalt structure, while the TX_2_ layer displays hexagonal symmetry, with metal atoms surrounded by six chalcogen atoms in either octahedral or trigonal prismatic coordination. The rocksalt MX layer in MLC tends to adjust its lattice parameters to adapt to the hexagonal structure of the TX_2_ layer, approximately to √3 × *a* of the TX_2_ layer. Therefore, the in-plane (*ab*) lattice parameters of the TX_2_ subunit can be conveniently indexed using an orthorhombic pseudohexagonal unit cell with dimensions *a*, *b* = *a*, √3 × *a*.

## Supporting Information Available

Sample description, electron microscopy images and analysis, XRD of time and temperature series, TEM and SEM images of annealed samples, FIB cross sections, and schematic of synchrotron experiment (PDF)
