# Supplementary material for: Nanotubes from Lanthanide-Based Misfit-Layered Compounds: Understanding the Growth, Thermodynamic, and Kinetic Stability Limits
Source: Chem Mater. 2024 Apr 30;36(9):4736–49. doi: 10.1021/acs.chemmater.4c00481 (PMC11104483; doi:10.1021/acs.chemmater.4c00481)
Supplement: Supplementary file 1 — cm4c00481_si_001.pdf [file cm4c00481_si_001.pdf]

Supporting information for

Nanotubes from Lanthanide based Misfit Layered Compounds: Understanding the Growth, Thermodynamic and Kinetic Stability Limits

M.B. Sreedhara,[1*#] Azat Khadiev,[2#] Kai Zheng,[3] Simon Hettler,[4,5] Marco Serra,[6] Ivano E. Castelli,[3] Raul Arenal,[4,5,7] Dmitri Novikov,[2] and Reshef Tenne[8*]

[1]Solid State and Structural Chemistry Unit, Indian Institute of Science, Bengaluru, 560012 India
[2]Deutsches Elektronen-Synchrotron DESY, Notkestr. 85, 22607 Hamburg, Germany
[3]Department of Energy Conversion and Storage, Technical University of Denmark, DK-2800 Kgs. Lyngby, Denmark
[4]Instituto de Nanociencia y Materiales de Aragon (INMA), CSIC-Universidad de Zaragoza, 50018 Zaragoza, Spain
[5]Laboratorio de Microscopias Avanzadas (LMA), Universidad de Zaragoza, 50018 Zaragoza, Spain
[6] Dipartimento di Scienze Chimiche e Geologiche, Università di Modena e Reggio Emilia, Via G. Campi 103, 41125 Modena, Italy
[7]ARAID Foundation, 50018 Zaragoza, Spain
[8]Department of Molecular Chemistry and Materials Science, Weizmann Institute of Science, Rehovot 7610001, Israel

# contributed equally
*corresponding authors: sreedhara@iisc.ac.in, reshef.tenne@weizmann.ac.il

Table of contents

1. Description of the samples $(SmS)_{1.19}TaS_2$ (**Table S1**)
2. Characterization of nanotubes
    2.1. X-ray diffraction measurements
    2.2. Scanning and transmission electron microscopy and EDS
3. Supplementary figures **S1 to S14**
4. Lattice strain estimation in $(SmS)_{1.19}TaS_2$ and $(LaS)_{1.14}TaS_2$

1. Description of the prepared $(SmS)_{1.19}TaS_2$ and $(LaS)_{1.14}TaS_2$ samples

Table S1. Description of various reaction parameters, temperature, time and transport agent for $(SmS)_{1.19}TaS_2$ MLCs nanotubes preparation and post annealing.

Number	Sample name.	Reaction Temperature (in °C)	Reaction time (in h)	Transport agent
	**Using different transport agents**			
1	$(SmS)_{1.19}TaS_2$	825 °C	4h	Cl
2	$(SmS)_{1.19}TaS_2$	825 °C	4h	Br
3	$(SmS)_{1.19}TaS_2$	825 °C	4h	I
4	$(SmS)_{1.19}TaS_2$	825 °C	4h	No transport agent
	**Temperature-dependence reactions**			
5	$(SmS)_{1.19}TaS_2$	800 °C	4h	Cl
6	$(SmS)_{1.19}TaS_2$	825 °C	4h	Cl
7	$(SmS)_{1.19}TaS_2$	850 °C	4h	Cl
8	$(SmS)_{1.19}TaS_2$	875 °C	4h	Cl
9	$(SmS)_{1.19}TaS_2$	900 °C	4h	Cl
10	$(SmS)_{1.19}TaS_2$	925 °C	4h	Cl
11	$(SmS)_{1.19}TaS_2$	975 °C	4h	Cl
	**Time-dependent reactions**			
12	$(SmS)_{1.19}TaS_2$	825 °C	1h	Cl
13	$(SmS)_{1.19}TaS_2$	825 °C	2h	Cl
14	$(SmS)_{1.19}TaS_2$	825 °C	4h	Cl
15	$(SmS)_{1.19}TaS_2$	825 °C	8h	Cl
16	$(SmS)_{1.19}TaS_2$	825 °C	16h	Cl
	**Annealed $(SmS)_{1.19}TaS_2$ MLC nanotubes prepared at 825 °C**			
17	$(SmS)_{1.19}TaS_2$	1050 °C	4h	Cl
18	$(SmS)_{1.19}TaS_2$	1150 °C	4h	Cl
19	$(SmS)_{1.19}TaS_2$	1150 °C	24h	Cl
20	$(SmS)_{1.19}TaS_2$	1200 °C	96h	Cl
	**Miscellaneous reaction conditions**			
21	$(SmS)_{1.19}TaS_2$	825 °C (single step)	4h	Cl
22	$(SmS)_{1.19}TaS_2$	825 °C (quenched to liquid nitrogen)	4h	Cl
23	$(SmS)_{1.19}TaS_2$	825 °C (slow cooling rate, 3 °C /min)	4h	Cl
24	$(SmS)_{1.19}TaS_2$	825 °C (slow heating and cooling rate, 3 °C /min)	4h	Cl
25	$(SmS)_{1.19}TaS_2$	1050 (single zone)	4h	Cl

2. Characterization of the nanotubes

2.1. Scanning and transmission electron microscopy (SEM & TEM) and chemical analysis (EDS):

Scanning electron microscopy (SEM) imaging was done with a Zeiss Sigma 500 model. A minute quantity of native sample was picked up by a capillary tube and dispersed on carbon tape for the SEM analysis. Energy dispersive X-ray spectroscopy analysis (EDS) was performed with the Bruker XFlash/60mm retractable detector. The relative abundance (yield) of the nanotubes was estimated by analyzing a number (at least 8-10 images were counted for each sample) of SEM images of the product. ImageJ software (26) has been used for the analysis of the nanotubes' abundance and their size distribution. The determined abundancies were based on counting the number of nanotubes and flakes in each image and dividing the number of nanotubes by the total number of nanotubes and flakes. The abundance was averaged over the number of images counted and an error bar was drawn. Similarly, the diameter of the nanotubes (> 100 tubes in each case) was measured using ImageJ software by calibrating the scale in the image. The abundance of nanotubes (number of nanotubes with a given diameter in the total number of nanotubes analyzed) was plotted as a function of the diameter. The relative abundance (yield) of the NT was estimated by analyzing many SEM images of the product. The determined yields were based on the relative surface area occupied by the NTs compared to the area occupied by the entire product in the SEM images. Each material was synthesized three times and at least ten SEM images for each product were analyzed. While being only semi-quantitative in nature, the overall yield did not vary appreciably from one batch (of the same material) to the other and the relative abundance of the nanotubes for the different MLCs was found to be reproducible.

Transmission electron microscopy (TEM) and selected area electron diffraction (SAED) patterns analyses were performed using a JEOL JEM2100 microscope operated at 200 kV. The analysis of the TEM images, including intensity profiles along the *c*-axis, and the SAED was performed with Digital Micrograph 3.1.0 (Gatan) software.

Two aberration-corrected (probe & image, respectively) Titan microscopes (Thermo Fisher Scientific) were used to perform high-resolution (HR) transmission electron microscopy (TEM) and scanning (S)TEM imaging using high-angle annular dark field (HAADF), annular dark field (ADF) and bright field (BF) detectors. The microscopes were operated at 300 kV and EDS was applied using the probe-corrected microscope equipped with a high-brightness field emission gun (X-FEG) and an EDAX Si(Li) detector. SAED patterns were additionally acquired using the image-corrected Titan microscope.

For HRSTEM experiments, the sample powders stored under vacuum were dispersed in ethyl alcohol. For each session, samples were freshly prepared by ultrasonication of the dispersion and drop casting of 2 µl of the dispersion onto holey carbon TEM copper grids (Quantifoil). To prevent sample contamination under the electron beam, a 14 s plasma cleaning step was implemented prior to insertion in the microscope. STEM-EDS data was analyzed using the Thermo Fisher Scientific Velox and quantified with the S-K, Sm-L and Ta-L edges.

X-ray diffraction measurements:

X-ray diffraction (XRD) measurements on powders were performed using TTRAX III (Rigaku, Tokyo, Japan) theta-theta diffractometer. The set-up was equipped with a rotating copper anode

X-ray tube operating at 50 kV/200 mA. The powders were spread on a zero-background Si holder and pressed with a glass to flatten the surface. A bent graphite monochromator and a scintillation detector were aligned to the diffracted X-ray beam. They were scanned in specular diffraction ($\vartheta/2\vartheta$ scans) from 3-80° (2ϑ) with a step size of 0.02° and a scan rate of 0.5° per min in Bragg-Brentano mode with variable slits. The XRD data was analyzed using JADE Pro software and PDF-4+ 2020 database (ICDD).

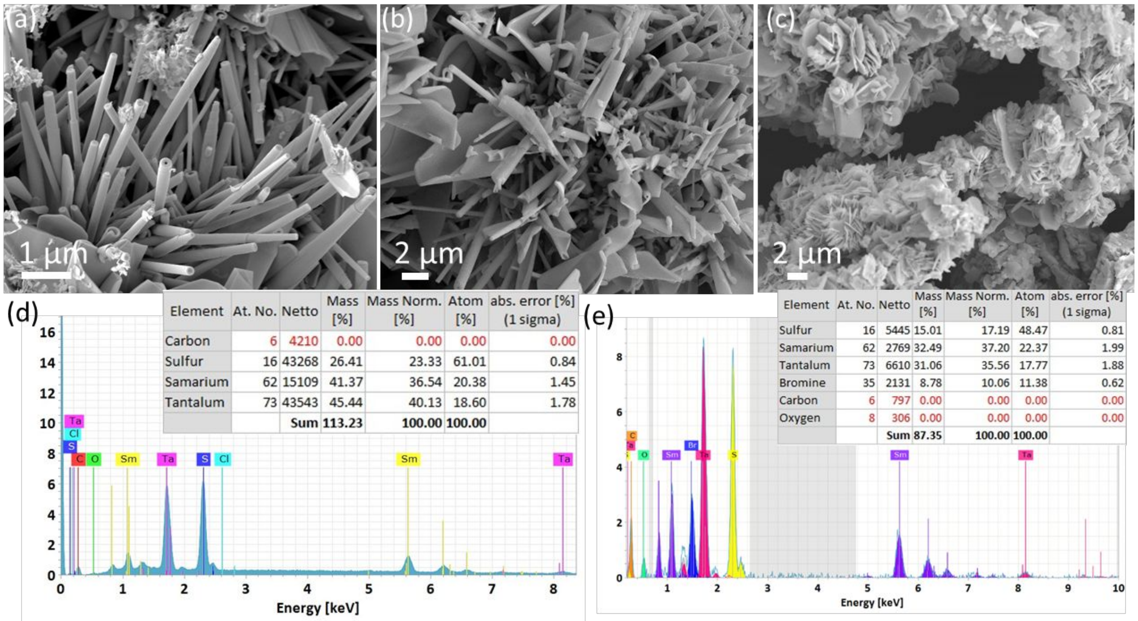

Element	At. No.	Netto	Mass [%]	Mass Norm. [%]	Atom [%]	abs. error [%] (1 sigma)
Carbon	6	4210	0.00	0.00	0.00	0.00
Sulfur	16	43268	26.41	23.33	61.01	0.84
Samarium	62	15109	41.37	36.54	20.38	1.45
Tantalum	73	43543	45.44	40.13	18.60	1.78
		Sum	113.23	100.00	100.00	

Element	At. No.	Netto	Mass [%]	Mass Norm. [%]	Atom [%]	abs. error [%] (1 sigma)
Sulfur	16	5445	15.01	17.19	48.47	0.81
Samarium	62	2769	32.49	37.20	22.37	1.99
Tantalum	73	6610	31.06	35.56	17.77	1.88
Bromine	35	2131	8.78	10.06	11.38	0.62
Carbon	6	797	0.00	0.00	0.00	0.00
Oxygen	8	306	0.00	0.00	0.00	0.00
		Sum	87.35	100.00	100.00	

Fig. S1. (a-c) SEM micrographs of the $(SmS)_{1.19}TaS_2$ MLC structures obtained using Cl, Br, and I as transport agents at 825 ℃, respectively (**Table 1** entry 1-3). It is evident that iodine does not yield nanotubes. (d, e) EDS chemical analysis of the $(SmS)_{1.19}TaS_2$ nanotubes obtained using Cl and Br as transport agents respectively. Please note the disparity in the %Cl and %Br (> 11 at%) in the nanotubes. (EDS reference lines: Cl K_{α}=2.6 keV, Br L_{α}=1.48021)

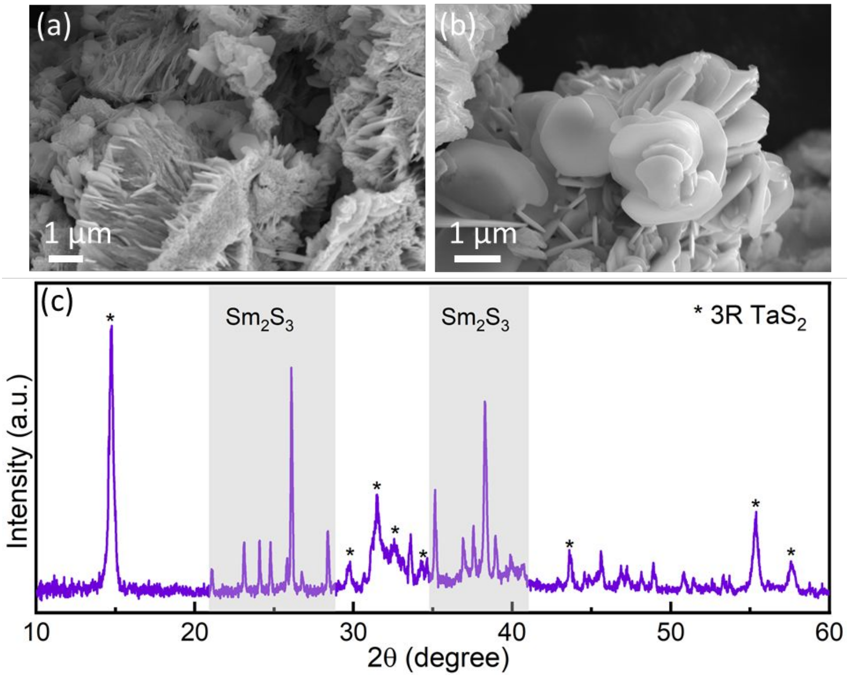

Fig. S2. (a-b) SEM micrographs of the reaction product obtained without using a transport agent (see **Table 1,** entry 4), the platelets structure corresponds to TaS_2 and the other phase comprising of Sm_2S_3. (b) XRD of the product showing the mixture of hexagonal 3R-TaS_2 and orthorhombic Sm_2S_3 phases confirming that misfit was not formed without transport agent with established reaction protocol. The major reflections from TaS_2 and Sm_2S_3 were identified.

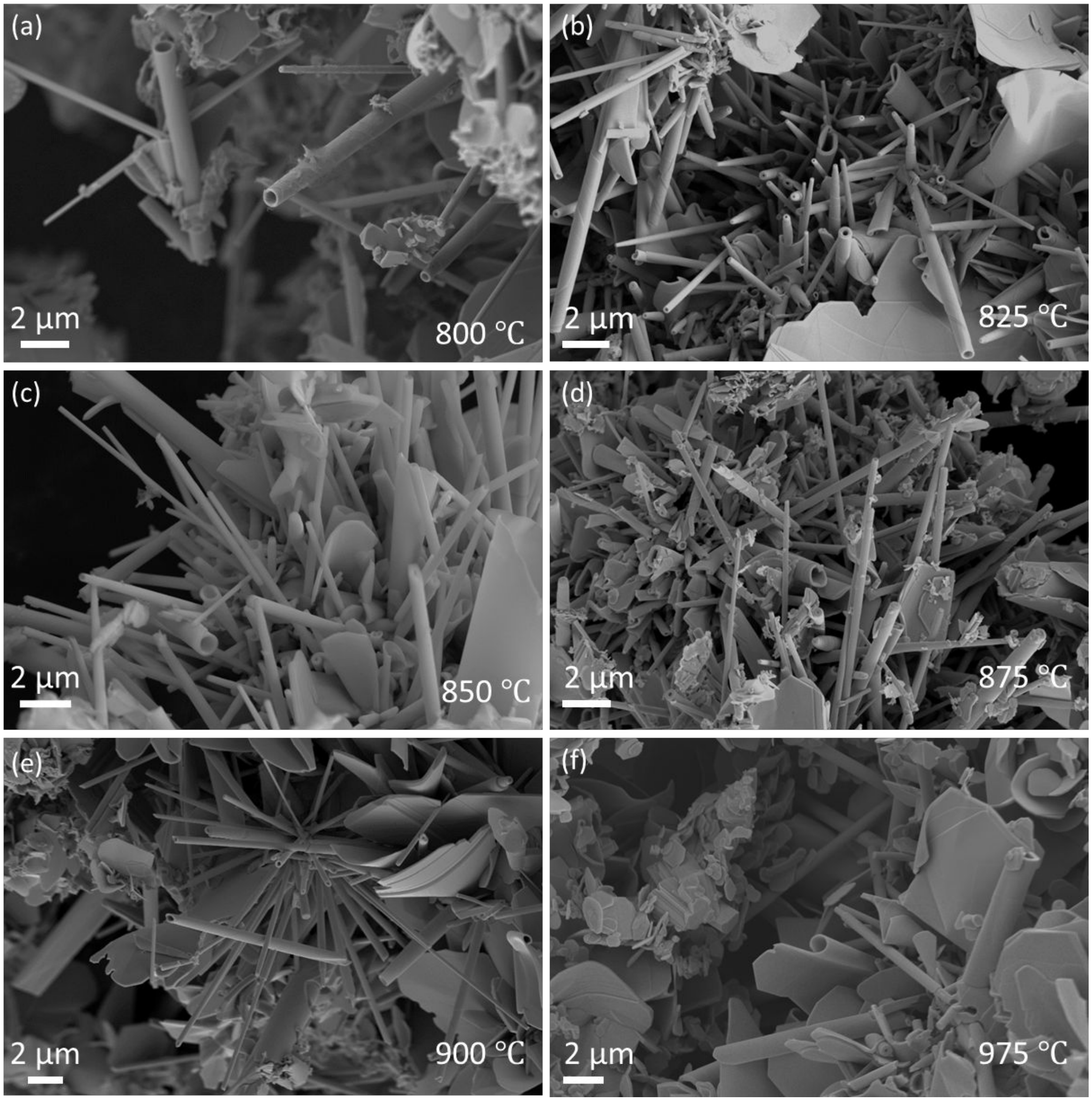

Fig. S3. SEM images of the $(SmS)_{1.19}TaS_2$ samples prepared at different temperatures from 800 ℃ to 975 ℃ for the reaction duration of 4 h. The variation of abundance and the aspect ratio of the nanotubes is visible.

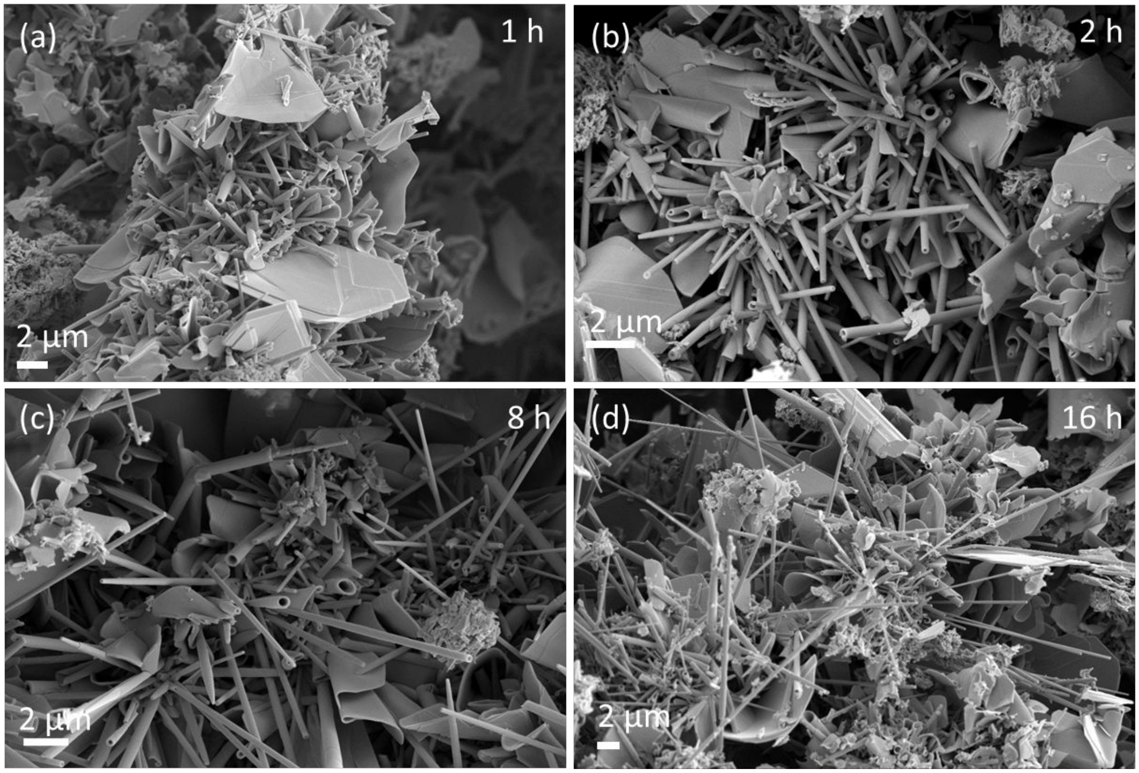

Fig. S4. SEM images of the $(SmS)_{1.19}TaS_2$ samples prepared at 825 ℃ at different reaction duration from 1 h to 16 h.

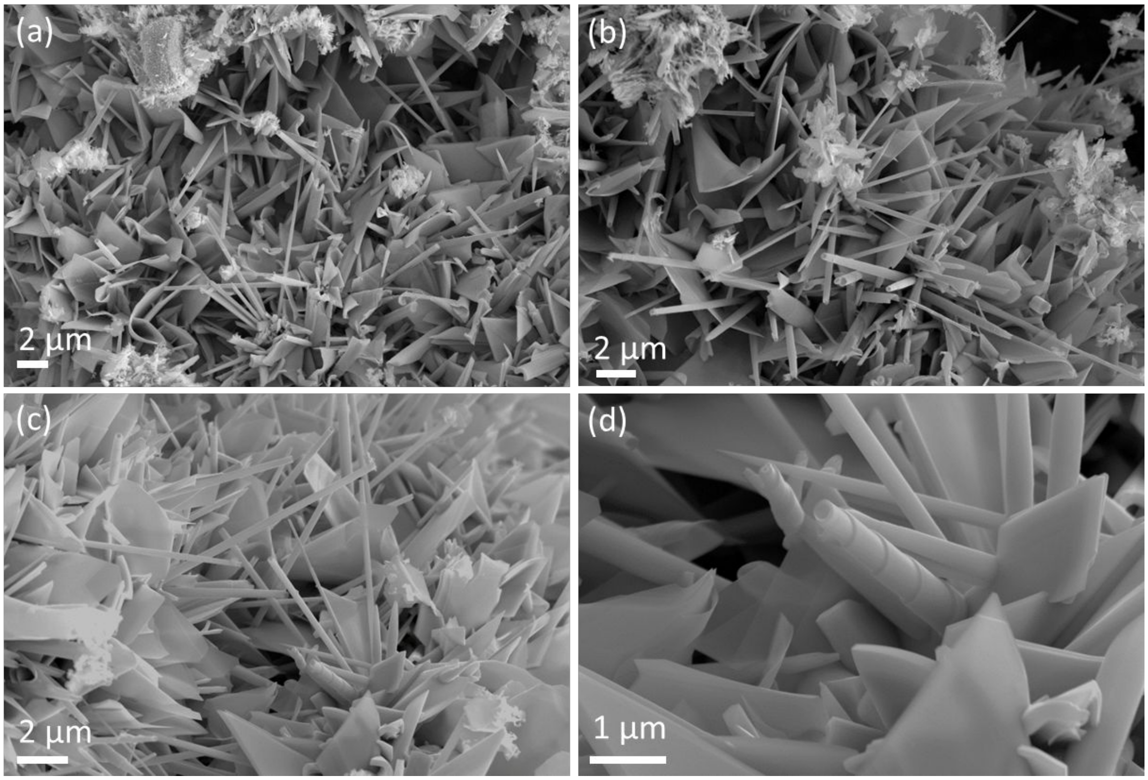

Fig. S5. SEM images of the $(SmS)_{1.19}TaS_2$ prepared at 825 ℃ and quenched into liquid nitrogen (LN_2).

Lattice strain estimation in $(SmS)_{1.19}TaS_2$ and $(LaS)_{1.14}TaS_2$

Rocksalt LaS, a= 5.813 Å
Rocksalt SmS, a=5.570 Å
Hexagonal 2H-TaS_2, a=3.314 Å

Approximate MLC unit in orthohexagonal cell $4a_{MX} \approx 7a_{TX2}$

% lattice strain between the layers in a-direction= $(7a_{TX2} - 4a_{MX})/7a_{TX2})*100$
(i.e., circumference of nanotube)

Without considering approximation: by formula unit
For LaS on TaS_2 = (2×3.314 - 5.813)/ 2×3.314 *100
= **12.3 %**
For SmS on TaS_2 = (2×3.314 - 5.570)/ 2×3.314 *100
= **16.00 %**

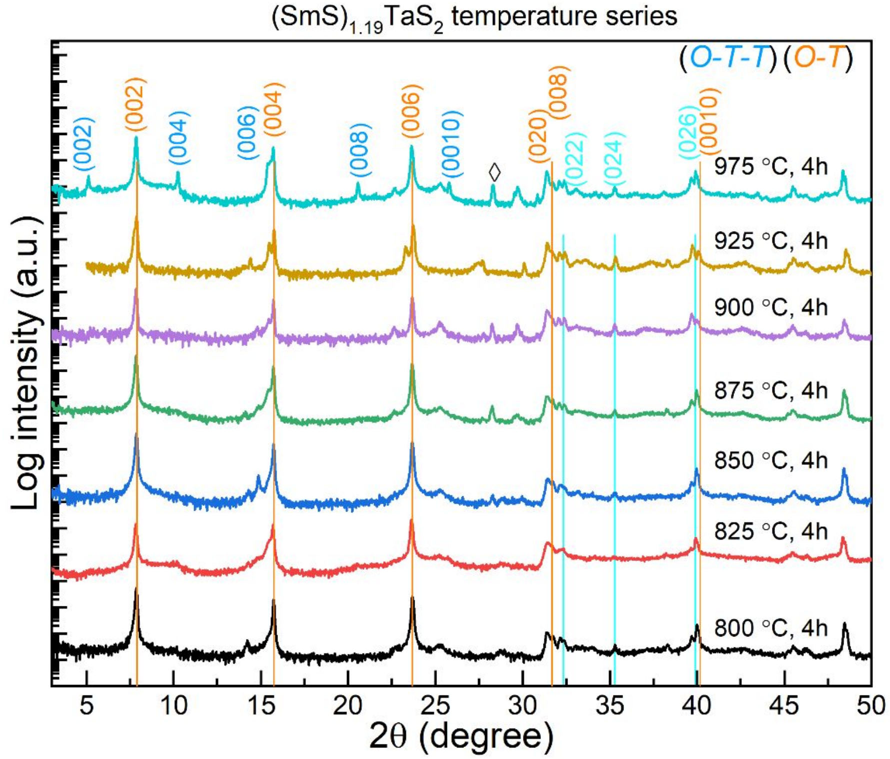

Fig. S6. Powder XRD patterns of $(SmS)_{1.19}TaS_2$ temperature series: The (00*l*) Bragg reflections associated with the (*O*-*T*) and (*O*-*T*-*T*) superstructures with *c*-axis periodicity 11.2 Å and 17.2 Å, respectively, are marked with orange and blue color, respectively. The peaks marked in diamond symbol in the range 28° in high temperatures and time correspond to cubic(I) Sm_2O_3 (PDF 04-004-8958)/ orthorhombic $Sm_2Ta_3S_2O_8$ phases (PDF 04-011-1697).

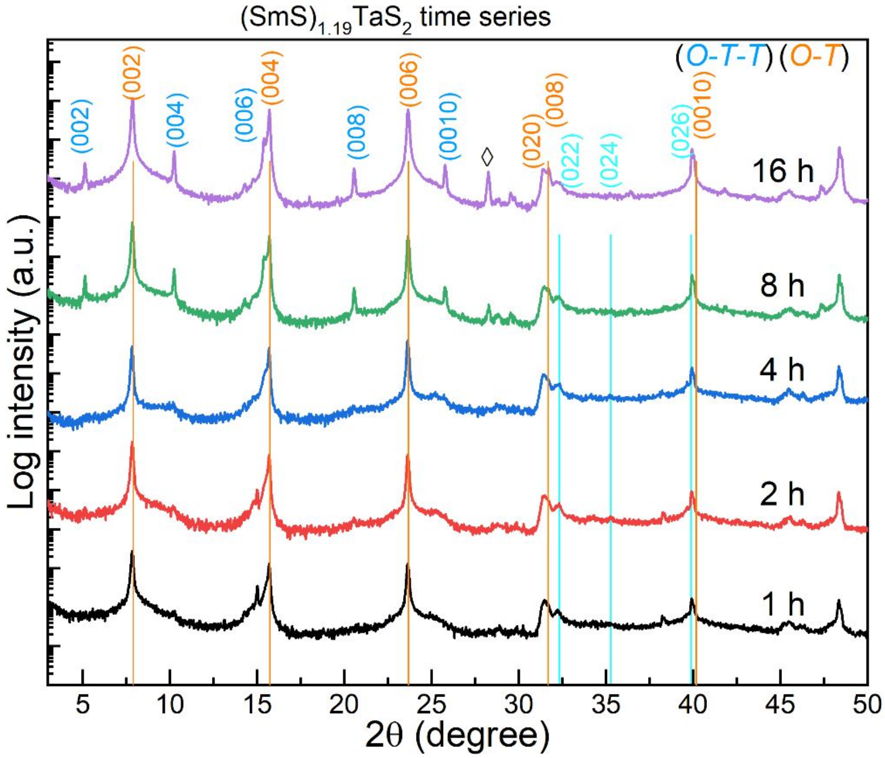

Fig. S7. Powder XRD patterns of $(SmS)_{1.19}TaS_2$ time series: The (00*l*) Bragg reflections associated with the (*O*-*T*) and (*O*-*T*-*T*) superstructures with *c*-axis periodicity 11.2 Å and 17.2 Å, respectively, are marked with orange and blue color, respectively. The peaks marked in diamond symbol in the range 28° in high temperatures and time correspond to cubic(I) Sm_2O_3 (PDF 04-004-8958)/ orthorhombic $Sm_2Ta_3S_2O_8$ phases (PDF 04-011-1697).

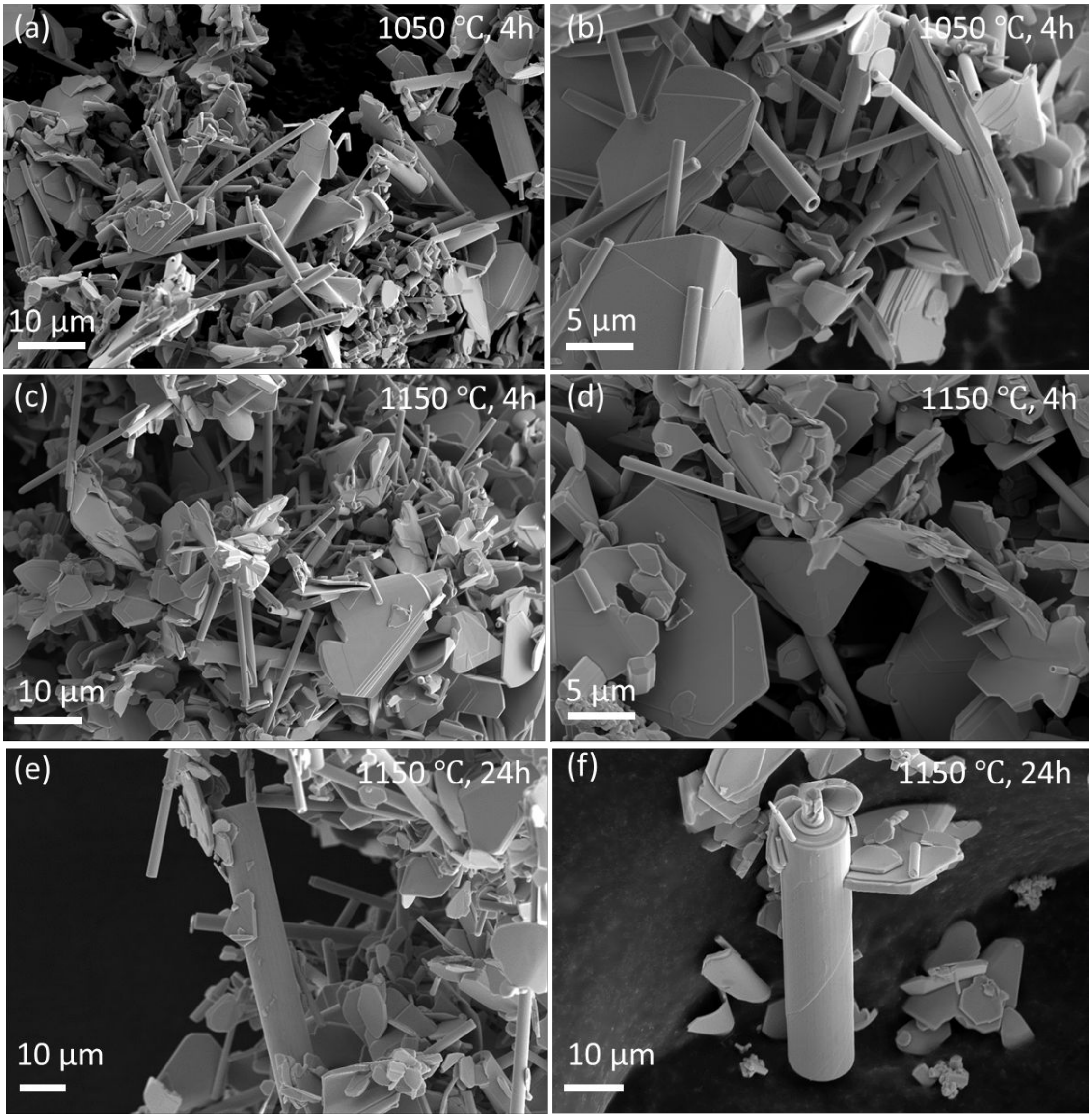

Fig. S8. SEM images of the $(SmS)_{1.19}TaS_2$ post annealed samples at different temperatures and times.

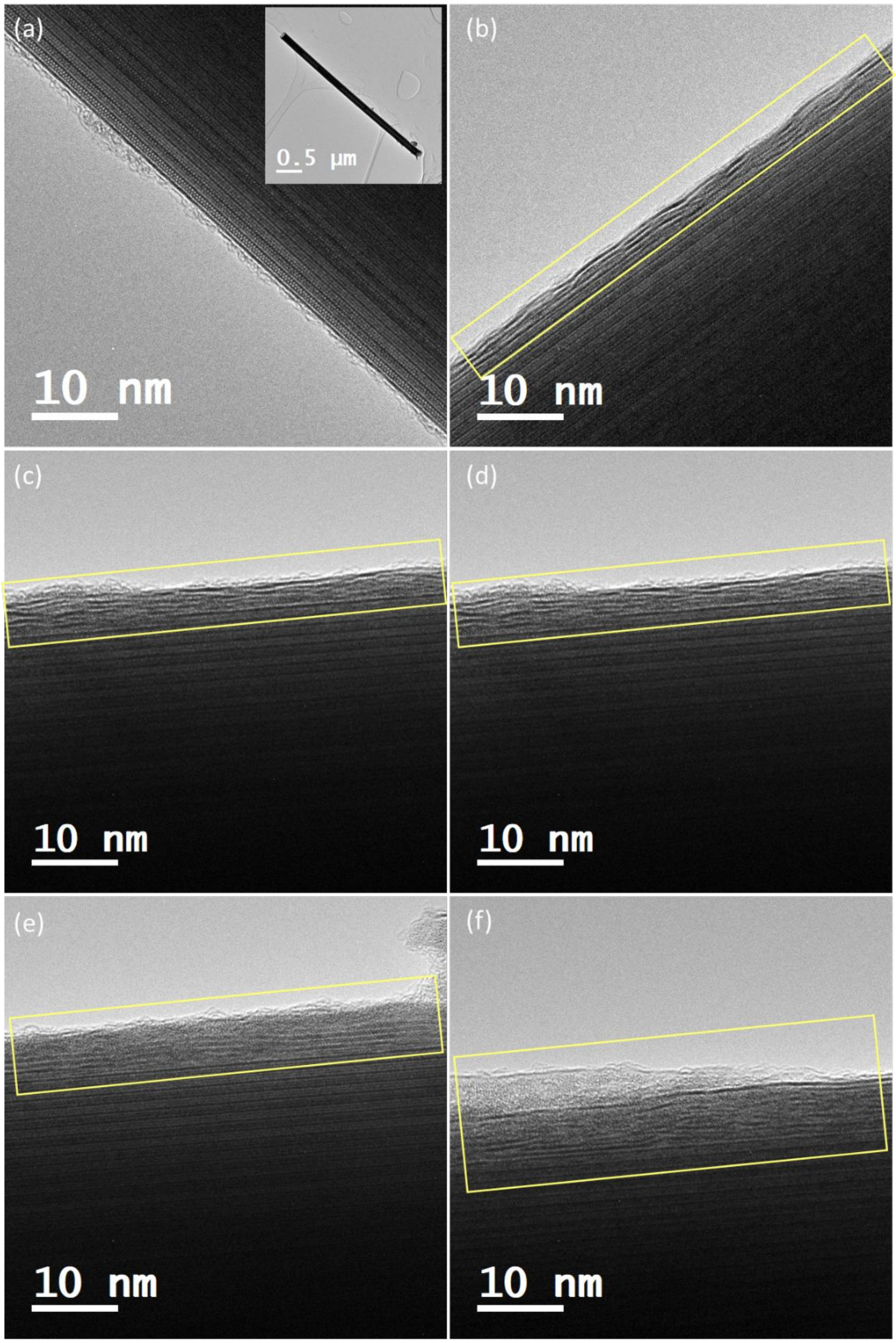

Fig. S9. High resolution TEM image of the $(SmS)_{1.19}TaS_2$ samples prepared at (a) 825 °C, 4h, showing the almost clean surface with the topmost layer being TaS_2. Subsequent images (b-f) show samples annealed at high temperatures. The surface of these nanotubes is highly modulated and shows discontinuous layers. The area in yellow rectangle shows the ruptures in the structures.

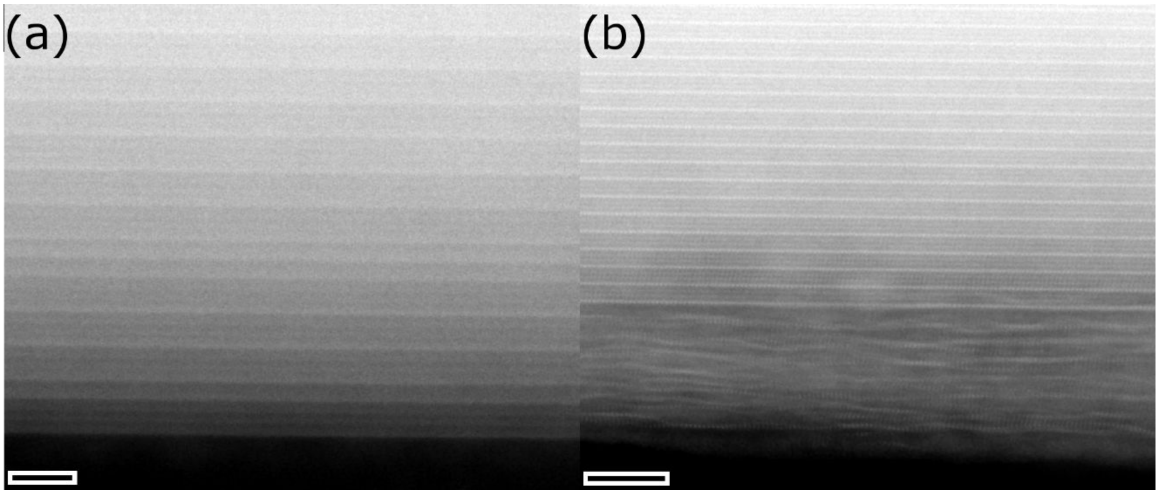

Fig. S10. HAADF-STEM images of the border of two $(SmS)_{1.19}TaS_2$ nanotubes annealed at (a) at 1050 °C and (b) at 1150 °C with transport agent showing an (*O*-*T*) and (*O*-*T*-*T*) stack in (a) and a disorder boundary in (b). Scale bars are (a) 2 nm and (b) 5 nm.

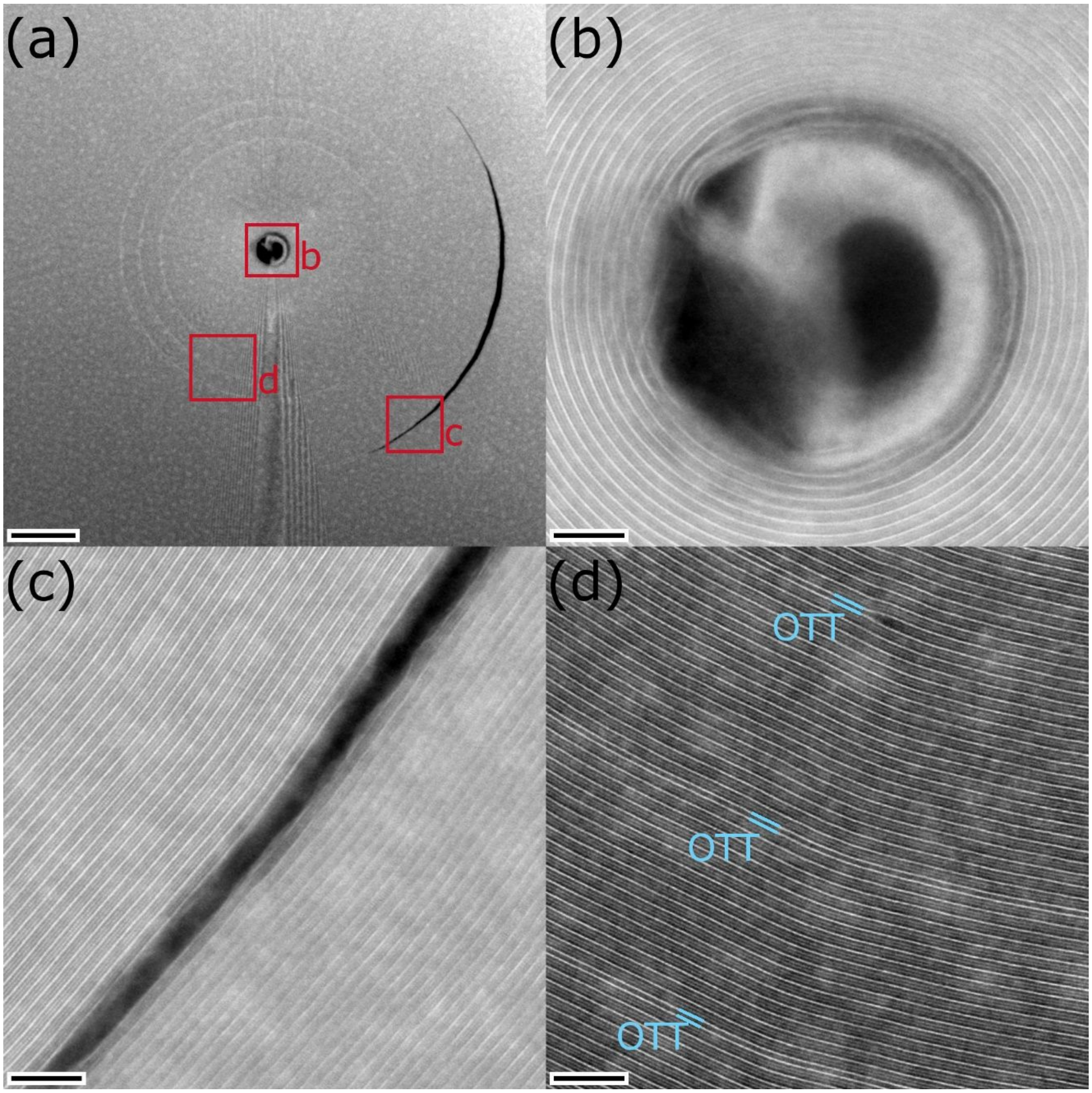

Fig. S11. HAADF-STEM images of a cross-section lamella prepared from a $(SmS)_{1.19}TaS_2$ tube (~500 nm) annealed at 1150 ℃ for 4h, by focused ion beam (FIB). (a) Overview of the inner part of the tube (scroll), the positions of images b, c and d are marked. (b) The central hollow core (35 nm diameter). The decomposition of the inner surface of the scroll is clearly visible and is consistent with previous images of the nanotube surface (Figs. S9 and S10). (c) On the upper right side, a gap is observed, possibly caused by de-intercalation/decomposition of SmS layer. (d) Three (*O*-*T*-*T*) layers are visible (marked in cyan) and they indicate the gradual decomposition of the (*O*-*T*) superstructure into (*O*-*T*-*T*). Scale bars are (a) 70 nm and (b-d) 7 nm.

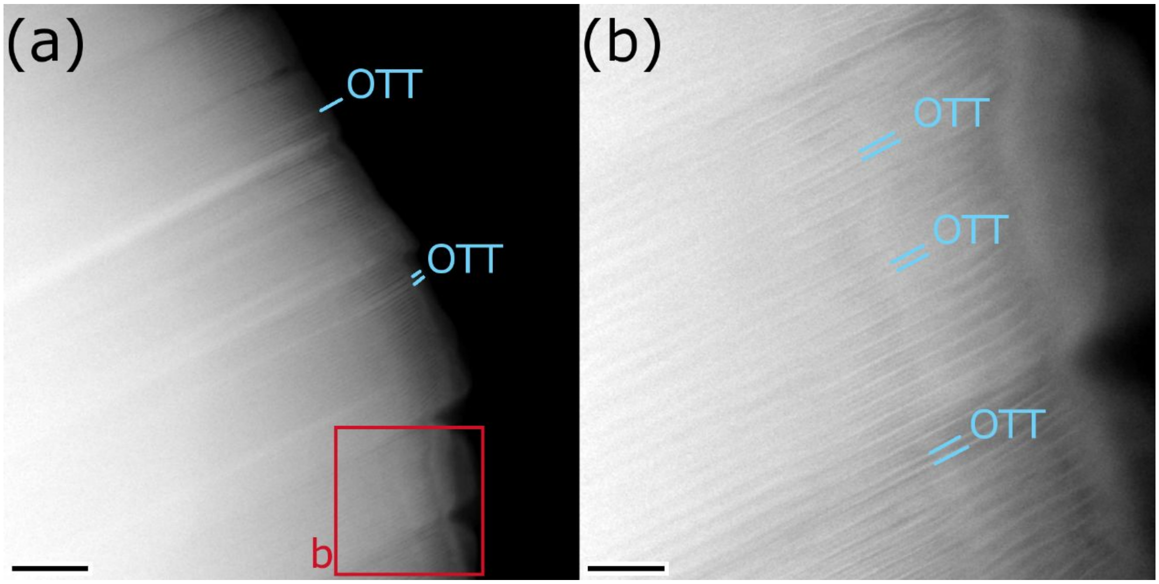

Fig. S12. HAAD-STEM images of the edge of a $(SmS)_{1.19}TaS_2$ flake annealed at 1150 °C. (a) Several (*O*-*T*-*T*) layers are observed already at lower magnification, (b) which are more clearly resolved at higher magnification. Scale bars are (a) 20 nm and (b) 5 nm.

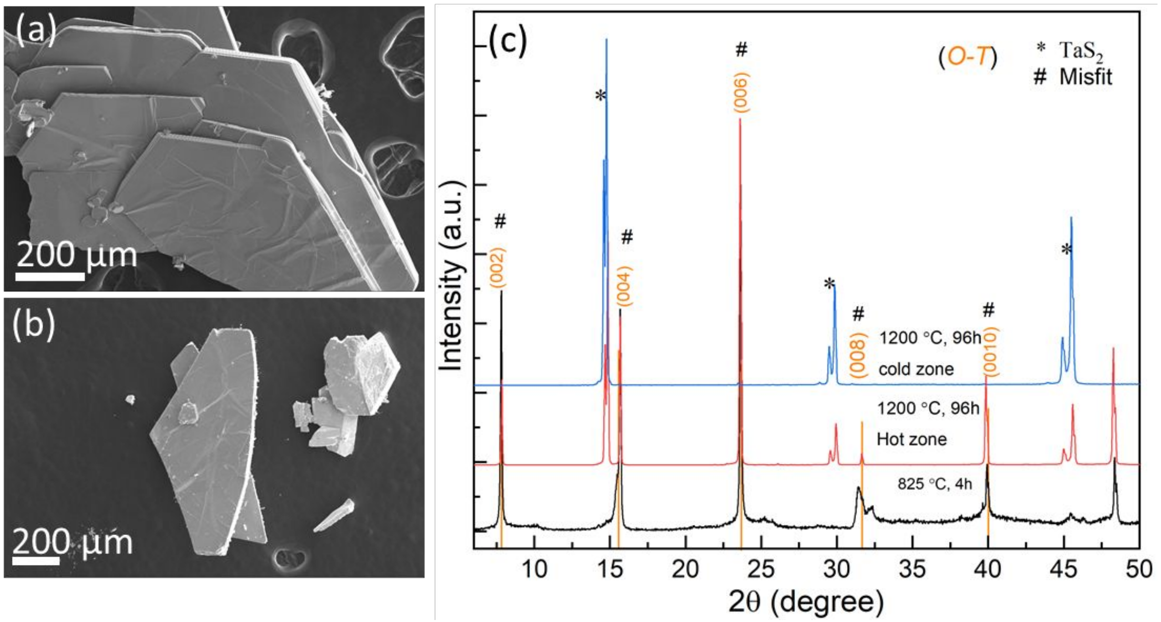

Fig. S13. SEM and XRD of the $(SmS)_{1.19}TaS_2$ nanotubes prepared at 825 °C (see Fig. 2b) and annealed at 1200 °C for 96 h. All the nanotubes converted into flakes. (a) SEM image of the flakes found at the hot zone and cold zone (b) respectively. (C) XRD patterns of annealed samples from hot zone (red) and cold zone (blue) in comparison with pristine sample. The flakes at the colder zone are show reflection corresponds to TaS_2. The flakes crystallized hotter zone comprise of mixture of TaS_2 (major) and misfit $(SmS)_{1.19}TaS_2$ (minor) phase.

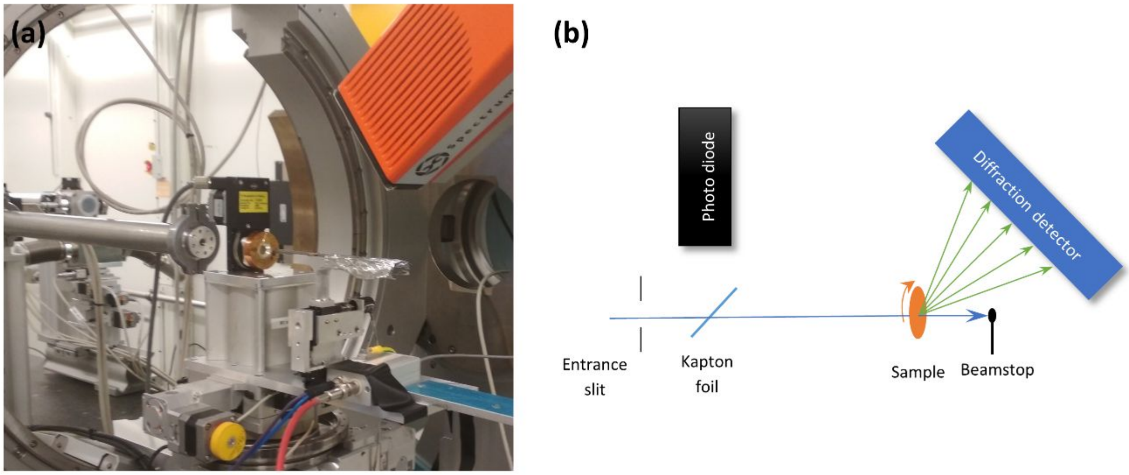

Fig. S14. Schematic representation of the experimental set-up used for the synchrotron powder XRD measurements: (a) photo of the setup, (b) setup scheme.
